# A reevaluation of selected mortality risks in the updated NCI/NIOSH acrylonitrile cohort study

**DOI:** 10.3389/fpubh.2023.1122346

**Published:** 2023-04-06

**Authors:** Gary M. Marsh, Adam Kruchten

**Affiliations:** Center for Occupational Biostatistics and Epidemiology, Department of Biostatistics, Graduate School of Public Health, University of Pittsburgh, Pittsburgh, PA, United States

**Keywords:** acrylonitrile, cancer, cohort study, mortality, industrial workers, bladder cancer, lung cancer, mesothelioma

## Abstract

**Objectives:**

The study aimed to determine whether the National Cancer Institute's (NCI) recent suggestion of associations between acrylonitrile (AN) exposure and mortality in lung and bladder cancer and pneumonitis is robust to alternative methods of data analysis.

**Materials and methods:**

We used the Richardson method to indirectly adjust risk ratios (RRs) in relation to AN exposure for potential confounding by smoking and asbestos. We repeated key analyses omitting workers from Plant 4 to account for possible local, historical shipyard-related asbestos exposures.

**Results:**

The adjustment of lung cancer RRs for confounding by both smoking and asbestos and omitting Plant 4 workers yielded mostly decreased RRs and much less evidence of a positive association with cumulative AN exposure.

**Conclusion:**

Overall, our reanalysis provided little evidence to support NCI's suggestion of associations between AN exposure and mortality in lung and bladder cancer and pneumonitis.

## Introduction

Acrylonitrile (AN) is an important industrial chemical used in the manufacturing of acrylic and modacrylic fibers, resins, plastics, elastomers, adiponitrile, and nitrile rubber for various consumer goods. The primary routes of potential human industrial exposures to AN are inhalation and dermal contact. Potential non-industrial AN exposures arise from the burning of biomass (e.g., wildfires and fuel wood) and as a component of tobacco smoke (1). Experimental studies with rats exposed to AN by inhalation, drinking water, or gavage have produced tumors in the brain, Zymbal's gland, forestomach, and mammary gland (2). Tumors of the Harderian gland and forestomach were subsequently reported in mice exposed to gavage (3). Early epidemiological studies of AN-exposed workers revealed no clear and consistent evidence of an excess human cancer risk related to AN exposure. Although some studies reported slightly elevated relative risks for cancers of the lung, bladder, prostate, and CNS (central nervous system) (4–11), the findings were inconsistent and limited by study deficiencies such as the lack of exposure information, small study size, and inability to adjust adequately for potential confounding factors such as cigarette smoking and asbestos. In 1999, based on sufficient evidence in experimental animals and inadequate evidence in humans, the International Agency for Research on Cancer classified AN as a possible human carcinogen (12). Subsequent and other earlier studies (13–15), reanalyses of individual cohorts (16–18), reviews (19), and meta-analyses (20) have shown no consistent association between AN exposure and increased mortality risks based on any cancer site.

The NCI study originally reported by Blair et al. (5), which remains the largest cohort study to date, originally examined mortality patterns through 1989 among 25,460 employees from eight U.S. facilities that produced AN or used AN as feedstock in other manufacturing processes. The authors found no consistent evidence of an AN exposure–response relationship for lung cancer but reported a nonstatistically significant 1.5-fold excess of lung cancer in the highest quintile of cumulative exposure (5). Further analysis by deciles of cumulative exposure yielded similar results. The NCI study, which was updated in 2019 and is the basis of our reanalysis reported herein, extended mortality follow-up through 2011. The authors concluded that they found additional evidence of an association between AN exposure and lung cancer, as well as a possible increased risk for death due to bladder cancer and pneumonitis (21). Additional details of the NCI study are detailed in the following sections.

### Updated NCI study: Further details and objectives of reanalysis

In 2019, NCI investigators extended the mortality follow-up of the NCI cohort of 21 years through 2011 and updated the case–cohort study by imputing smoking histories for newly identified lung cancer deaths (21). External mortality comparisons revealed an isolated statistically significant overall excess in mesothelioma [Standardized Mortality Ratio (SMR) = 2.24, 95% CI = 1.39–3.42]. Internal mortality comparisons again revealed the largest lung cancer (defined as lung and bronchus cancer) excesses in the highest quintile of cumulative AN exposure [Hazard Ratio (HR) = 1.43, 95% CI = 1.13–1.81], with marginal evidence of an exposure–response relationship (trend *p*-value = 0.05). Mortality risks from bladder cancer (defined as urinary bladder cancer) and pneumonitis (defined as pneumonitis due to solids and liquids) were also the largest in the highest cumulative AN exposure category, and both causes of death revealed a statistically significant trend with increasing cumulative AN exposure. No exposure–response relationship was found for mesothelioma. The authors concluded that their findings provided additional evidence of an association between AN exposure and lung cancer, as well as possible increased mortality risks for bladder cancer and pneumonitis.

The assessment of the cause-specific mortality risks in both NCI reports was based almost exclusively on internal study group comparisons (5, 21). The strengths of the internal study group comparison are that it will usually reduce the healthy worker effect (22) and it allows direct comparison of relative risk across strata. However, internal comparisons can be unstable when the study population is small (producing wider confidence limits) and may be misleading if workers included in the baseline category (i.e., least exposed or unexposed) have different underlying cancer risks than workers in the exposed groups. On the contrary, external comparisons based on regional rates have the strength of being able to adjust to geographic variability in social, cultural, and economic factors related to disease (23) and are generally very stable. The disadvantages of the external comparison group are the inability to adjust to the healthy worker effect and the difficulty in comparing standardized mortality ratios between groups when their confounder distributions differ (24).

In 2001, Marsh et al. reported a reanalysis of the lung cancer risk using cohort data from the original NCI study (5) that focused on AN exposure-specific lung cancer rates *via* external mortality comparisons (25). The authors found that the internal comparison-based NCI lung cancer excess among the most highly AN-exposed workers arose because of a small deficit in lung cancer deaths in the highest AN exposure category (regional rate-based SMR = 0.92, 95% CI = 0.6–1.4) was compared to a large, statistically significant deficit in deaths among unexposed workers that served as the baseline for the internal comparisons (SMR = 0.68, 95% CI = 0.5–0.9). Based largely on this and similar findings (25), it was concluded that their reanalysis provided little evidence that AN exposure increases lung cancer risk.

Another key shortcoming of both NCI study reports was the limited adjustment of lung cancer risk estimates for potential confounding by smoking or asbestos, and in the latest report, no adjustment of bladder cancer risk estimates was seen for potential confounding by smoking. That is, while the NCI case–cohort study enabled lung cancer risk estimates for 10% of the cohort (along with imputed smoking histories for new cases) to be adjusted for potential confounding by smoking, it did not permit smoking adjustment at the cohort level. Furthermore, based on unreasonable relative risk estimates for smoking and lung cancer, the smoking data in the original NCI study were likely to be misclassified (26) (Cunningham, MS, unpublished thesis, University of Pittsburgh, 2005). Adjustment for asbestos in the latest NCI study was based on semi-quantitative asbestos exposure probability scores.

We report here our re-analysis of the cohort data from the 2011 NCI cohort update and compare our findings to those of Koutros et al. (21) We focused on those findings as follows: (1) The relationship between cumulative AN exposure and mortality from the four causes of death categories of *a priori* interest: lung cancer, bladder cancer, mesothelioma, and pneumonitis using external comparisons (SMRs); and (2) indirect adjustment for potential confounding by smoking (lung and bladder cancer) and asbestos (lung cancer) at the cohort level and within AN exposure categories. We repeated key analyses omitting workers from one study plant (Plant 4) to account for possible historical shipyard-related asbestos exposures in the local area.

## Methods

### Reconstruct cohort data file

We obtained a copy of the updated NCI AN study data from the authors. This file included individual demographic, work history, and AN exposure data for 25,460 workers. Further details about the NCI-NIOSH study are provided by Blair et al. (5) and Koutros et al. (21). Due to the summary nature of the NCI work history and AN exposure data, we first reconstructed a cohort analysis file that was compatible with the more detailed input format of the OCMAP-Plus cohort analysis program (27). Because of the many assumptions required to reformat the NCI cohort file, we performed an extensive cross-check to establish the comparability of the NCI and our cohort data. Appendix A provides details of the study file reformatting and validation process. To comply with the data transfer agreement between the University of Pittsburgh and the NCI, no cohort data used or generated in our reanalysis were disclosed for any subcohort unit smaller than 10 subjects.

### External mortality comparisons

We computed standardized mortality ratios (SMRs) and 95% confidence intervals (CI) for the four *a priori* causes of death categories among AN-exposed and unexposed workers stratified by study plant and in all plants combined. The four *a priori* categories were defined by the same International Classification of Diseases (ICD) codes used by NCI (21). SMRs were also computed for the categories (quintiles) of cumulative AN exposure (lagged 10 years as done by NCI) and time since the first AN exposure was reported in the NCI study. All SMRs were adjusted for race, sex, age group, and time period. As in the NCI study, person-year counts in the unexposed or lowest exposure baseline categories include the observation time of workers before their first AN exposure. SMRs were computed using both total U.S.-specific and plant-specific regional mortality rates developed from the Mortality Information and Research Analytics (MOIRA) program maintained at the University of Pittsburgh (27). Regional rates were based on an aggregate of individual counties or parishes from which at least 80% of each plant workforce resided. We show below the plant number, the counties or parishes comprising the regional rate, the 2,000 total populations (N) of the regional areas, and the total U.S. population in 2,000.

*Plant 1:* Santa Rosa and Escambia, FL (*N* = 412,153)
*Plant 2:* Brazoria, Galveston, and Harris, TX (*N* = 3,892,503)
*Plant 3:* Allen, Auglaize, Hardin, Hancock, Mercer, Putnam, and Van Wert, OH (*N* = 363,633)
*Plant 4:* Hampton City, Newport News City, and James City, VA (*N* = 374,689)
*Plant 5:* Morgan, Lawrence, Limestone, Cullman, and Madison, AL (*N* = 565,726)
*Plant 6:* Jefferson, Orleans, and St. Charles Parishes, LA (*N* = 988,212)
*Plant 7:* Hamilton, OH and Dearborn and Ripley, IN (*N* = 917,935)
*Plant 8*: Galveston and Brazoria, TX (*N* = 491,925)
*Total U.S*. (*N* = 281,421,906)

Our interpretation of the external comparisons focuses on the regional rates as we believe that they usually provide the most valid external comparison by helping to adjust for the social, cultural, and economic factors related to the disease. In addition, the large size of the regional populations shown earlier assures the stability of the associated death rates. We compared our U.S. and regional rate-based SMRs with the U.S. rate-based SMRs and internal rate-based hazard ratios (HRs) computed by Koutros et al. (21).

### Indirect adjustment for potential confounding at the cohort level

#### Negative control outcome confounding adjustment

We used the methods described by Richardson (28) and Richardson et al. (29) to indirectly adjust internal cohort relative risks [Risk Ratio (RRs)] for lung cancer, bladder cancer, and pneumonitis among AN-exposed workers for potential confounding by smoking. This cohort-level method provides a control for the effect of unmeasured smoking through a negative control or proxy outcome that is known to be caused by smoking but not caused by the exposure of interest. We chose the cause of death category, i.e., chronic obstructive pulmonary disease (COPD), as the smoking-related cause of the death category that is not associated with AN exposure (i.e., negative control outcome). We applied the same method to indirectly adjust RRs for lung cancer for potential asbestos exposure (and for both smoking and asbestos exposure), using the cause of death category mesothelioma as the negative control outcome. The NCI study provided no evidence that mesothelioma mortality risks were related to AN exposure. We applied the Richardson method to both categorical and continuous forms of the AN exposure data (continuous data limited to lung cancer and COPD).

The Richardson method requires some strong assumptions that cannot be verified from data but must be informed by outside knowledge. First, and most critically, is the negative control condition that stipulates that the exposure of interest (AN) has no causal effect on the negative control outcome (COPD). If the negative control condition is not satisfied, the method is not valid, and it is not clear what the adjusted coefficients may mean. The second assumption is the equi-confounding assumption that requires that the unobserved confounders should have the same overall effect on both the negative control outcome (COPD) and the outcome of interest (lung cancer). If the equi-confounding assumption is satisfied, the Richardson method is able to remove unobserved confounders. In the case that the equi-confounding assumption is not fully satisfied, it must be noted that the Richardson method only provides partial adjustment. We further note that if one believes that the equi-confounding is not approximately true, the adjustments provided by the Richardson method may still contain some residual confounding bias. In this case, one should be cautious in the interpretation of the adjusted coefficients but can still use them as indicators of the presence of uncontrolled confounders (30).

We fitted relative risk regression models of the form λ(*t*) = λ_0_(*t*) exp^**x**(*t*)β^ to the internal cohort rates (31–33). For fitting the models, we used the exact conditional logistic regression procedure in the survival package (34) for R 4.0 software (35). For each model, we estimated the parameters β and the associated RRs. For each cause of death, risk sets were explicitly constructed using a nested case–control design from the NCI cohort data using the RISKSET program module in OCMAP-PLUS (27). We matched risk sets on the exact age at death (event time) and year of birth (±1-year caliper). We included race, sex, and pay type (wage or salary) as covariates in the resulting conditional logistic regression models. Time-dependent AN exposures were evaluated for each study member at each event time they were at risk.

We point out that conditional logistic regression is used to fit the model due to the use of explicitly constructed risk sets and is equivalent to estimating a stratified Cox proportional hazard model where strata are possibly varying in time (36). Furthermore, we clarify that this model estimates RRs due to the use of the nested case–control design. Some readers may be concerned that the use of conditional logistic regression mean results must be interpreted as an odds ratio. However, due to the study design, the odds ratio estimated by the conditional logistic regression is an exposure odds ratio rather than a disease odds ratio. The exposure odds ratio in this design is exactly equal to a rate ratio of mortality, which can further be interpreted as an RR as death can only occur at most once per individual (37). We further point out that our method to compute internal RRs is analogous to the Cox regression modeling used by NCI (21) and produced consistent results. Additional mathematical details of our application of the Richardson method and associated variance estimates are provided in Appendix B.

#### Bias adjustment factors and Monte Carlo sensitivity analysis

We also applied the indirect method proposed by Miettinen (38) and described by Axelson and Steenland (39) and Steenland and Greenland (40) to adjust selected external mortality comparisons for lung and bladder cancer for potential confounding by smoking (full cohort for both and plant-specific for lung cancer—UPitt regional rate-based SMRs for workers in the highest cumulative AN exposure category). The basic approach to adjusting SMRs indirectly first requires determining the confounding risk ratio (CRR), which is a function of the estimated RR for smoking and lung cancer among the cohort and also the prevalence of ever smoking among cohort members and members of the corresponding external standard populations. From the NCI case–cohort study, we used the reported RR = 19.1 for ever smoking and lung cancer and computed the overall and plant-specific prevalence of ever smoking. For smoking and bladder cancer, we used an RR = 3.5 (41). Because smoking data for the newly identified lung cancer cases and some original cases were imputed by NCI and not included in the data file, we adjusted our plant-specific prevalence measures by a factor of 0.68/0.63 = 1.08^1^. We used the Behavioral Risk Factor Surveillance System Survey, 1995, (42) to determine smoking prevalence for the states that included each study plant. The “all plants” ever-smoking prevalence was reported by Koutros et al. (21) as 0.68.

Because the indirect method described earlier produces only a point estimate of the smoking-adjusted SMR, we applied Monte Carlo simulation-based sensitivity analysis to allow the inclusion of additional uncertainty by simulating plausible ranges of the unmeasured confounder variables and generating a distribution of possible parameter estimates and uncertainty values (40).

### Plant 4 sensitivity analysis

Plant 4, which includes 3,379 study members (13.3% of the cohort), is located in the area of the Newport News Shipyard, the largest and oldest naval shipbuilding site in the United States. Because Newport News used hundreds of asbestos-containing products to build ships, its shipyard workers have an elevated risk of asbestos-related diseases including lung cancer and mesothelioma (43–50). Given the possibility that some, if not many, of the Plant 4 study members had prior or subsequent employment at Newport News, we repeated some of our key external and internal mortality comparisons with Plant 4 subjects who were omitted or isolated to evaluate the extent to which these possible extraneous asbestos exposures may have impacted our overall results.

## Results

### Issues identified in the NCI cohort file and analysis

#### Cohort deaths in 2012

Our efforts to reformat and validate the NCI cohort file revealed that 42 cohort members had dates of death in 2012 despite the NCI-reported study end date of 31 December 2011. These dates occurred uniformly across the year 2012 and included seven deaths in two of the four *a priori* causes of death categories (five lung and bronchus cancers and two urinary bladder cancers). Because the total death and specific cause of death counts in the NCI and UPitt cohorts matched using the 2011 study end date, we assumed that the NCI analysis must have somehow included the 42 deaths that occurred in 2012 within the earlier 1942–2011 period. To examine the impact of not extending follow-up properly through 2012, we computed regional rate-based SMRs for all causes of death including lung and bladder cancer, using both a 2011 and 2012 study end date and compared these with the U.S. rate-based SMRs reported by NCI (Table 1).

**Table 1:** Observed deaths, NCI U.S. rate-based SMRs^a, b^, UPitt U.S. and regional rate-based SMRs^b^ for key causes of death by AN exposure, full NCI AN cohort, 1942–2011.

	**Lung and bronchus**		**Mesothelioma (ICD10 only)**		**Urinary bladder (underlying cause only)**		**Pneumonitis (solids/liquids)**	
	**Obs**	**SMR (95% CI)**	**Obs**	**SMR (95% CI)**	**Obs**	**SMR (95% CI)**	**Obs**	**SMR (95% CI)**
**Overall**								
NCI	808	0.87 (0.81–0.93)	21	2.24 (1.39–3.42)	55	0.84 (0.63–1.10)	27	0.66 (0.43–0.96)
UPitt (U.S.)	808	0.86 (0.80–0.92)	21	2.36 (1.46–3.61)^c^	55	0.80 (0.60–1.04)	27	0.53 (0.35–0.78)
UPitt (regional)	808	0.74(0.69–0.79)	21	1.66 (1.03–2.54)	55	0.81 (0.61–1.05)	27	0.52 (0.34–0.76)
**AN-Unexposed**								
NCI	249	0.84 (0.74–0.95)	d.s	d.s.	16	0.81 (0.46–1.31)	d.s.	d.s.
UPitt (U.S.)	249	0.83 (0.73–0.94)	d.s.	d.s.	16	0.77 (0.44–1.25)	d.s.	d.s.
UPitt (regional)	249	0.73 (0.64–0.82)	d.s.	d.s.	16	0.77 (0.44–1.25)	d.s.	d.s.
**AN-exposed**								
NCI	559	0.88 (0.81–0.96)	16	2.36 (1.35–3.83)	39	0.86 (0.61–1.17)	23	0.83 (0.53–1.24)
UPitt (U.S.)	559	0.88 (0.80–0.95)	16	2.48 (1.42–4.03)	39	0.82 (0.58–1.12)	23	0.67 (0.42–1.00)
UPitt (regional)	559	0.74 (0.68–0.80)	16	1.70 (0.97–2.76)	39	0.83 (0.59–1.13)	23	0.64 (0.41–0.96)

d.s. Data suppressed to comply with the NCI-UPitt data transfer agreement.

^a^From Koutros et al. (21) Table 2.

^b^SMRs adjusted for race, sex, age, and calendar time.

^c^With a time period at risk of 1942, and, later, the UPitt SMR matches the NCI SMR of 2.24. Adjusting to an ICD-10 revision, only the time period at risk gives the SMR of 2.36.

Supplemental Table 1 shows that for all causes of death categories, the UPitt SMRs based on both study end dates were uniformly less than the NCI U.S. rate-based SMRs. Moreover, the Upitt SMRs using the 2011 end date were ~4–5% greater than those based on the 2012 end date, suggesting that all NCIs 2011-based SMRs were inflated by this amount. The UPitt SMR for bladder cancer in the full cohort based on the 2012 end date revealed a statistically significant 25% deficit deaths (SMR = 0.76, 95% CI = 0.57–0.99). For the sake of consistency with the NCI analysis, we recorded the date of the death of the 2012 deaths to 31 December 2011 and used the same 2011 end date in all reanalyses.

#### Inconsistently reported follow-up periods

NCI reports that “Person-years of follow-up for SMRs were characterized from time of hire, the earliest of which was 1942, through 2011” (21). Given this, the titles of the NCI SMR tables, Table 2 and Web Table 5, are incorrect and misleading as they indicate a 1960–2011 follow-up, and Table 3 indicates a 1952–2011 follow-up. The earliest date of hire among cohort members was 1942, and the date of the first AN production was 1952. We identified 51 deaths between 1952 and 1959, and a very small number of person-years at risk (and no deaths) between 1942 and 1951. Thus, we found SMRs based on 1942–2011 to be very close to those based on 1952–2011, but for the sake of completeness and consistency, we chose to use 1942–2011 as the follow-up period for all external and internal mortality comparisons. NCI's use of a 1952–2011 follow-up for their internal analyses is consistent with the cohort data and reported accurately.

**Table 2:** Observed deaths, NCI internal rate-based HRs, and UPitt external rate-based SMRs (U.S. and regional rates) for selected causes of death by NCI cumulative acrylonitrile exposure category (lagged 10 years unless otherwise noted), full cohort, in the follow-up period 1942–2011.

**Cancer site cumulative exposure to acrylonitrile^a^**	**NCI internal rate analysis**			**UPitt analysis**		
				**Internal rates**	**External rates**	
	**Observed deaths**	**HR**^b^ **(95% CI)**	**Observed deaths**	**RR**^b^ **(95% CI)**	**U.S. rates SMR** ^c^ **Observed deaths (95% CI)**	**Regional rates SMR** ^c^ **Observed deaths (95% CI)**
**Cause of death categories showing elevated risks in NCI study**						
**Lung & bronchus cancer**						
Unexposed^d^	263	1	263	1	0.83 (0.73–0.93)	0.72 (0.64–0.81)
Exposed	545	not shown	545	1.08 (0.92–1.26)	0.88 (0.81–0.96)	0.74 (0.68–0.81)
0–0.09	109	1.15 (0.92–1.45)	109	1.12 (0.89–1.41)	0.90 (0.74– 1.08)	0.76 (0.62–0.92)
>0.09–0.64	109	0.96 (0.77–1.21)	111	0.96 (0.76–1.21)	0.75 (0.62– 0.91)	0.64 (0.52–0.77)
>0.64–2.30	109	1.03 (0.81–1.29)	107	1.00 (0.80–1.26)	0.83 (0.68–1.00)	0.70 (0.57–0.84)
>2.30–12.08	109	1.06 (0.84–1.33)	109	1.03 (0.82–1.30)	0.86 (0.70–1.03)	0.72 (0.59–0.87)
>12.08	109	1.43 (1.13–1.81)	109	1.42 (1.12–1.80)	1.17 (0.96–1.41)	1.00 (0.82–1.20)
*p*–trend		0.05		0.07	0.047	0.09
**Mesothelioma (ICD–10 only, 1999–2011)**						
Unexposed^d^	5	1	d.s.	1	d.s.	d.s.
Exposed	16	not shown	16	1.22 (0.43–3.44)	2.48 (1.42–4.03)	1.70 (0.97–2.76)
0–1.33	8	1.27 (0.40–4.01)	d.s.	d.s.	d.s.	d.s.
>1.33	8	1.15 (0.37–3.66)	d.s.	d.s.	d.s.	d.s.
p–trend		0.84		0.79	0.64	0.72
**Urinary bladder cancer**						
Unexposed ^d^	16	1	16	1	0.74 (0.42–1.19)	0.74 (0.42–1.20)
Exposed	39	not shown	39	0.91 (0.50–1.65)	0.83 (0.60–1.14)	0.84 (0.60–1.15)
0–0.37	13	0.94 (0.45–1.99)	12	0.80 (0.37–1.72)	0.74 (0.38–1.29)	0.74 (0.39–1.29)
>0.37–6.69	13	0.78 (0.37–1.65)	13	0.73 (0.35–1.55)	0.64 (0.34–1.10)	0.64 (0.34–1.10)
>6.69	13	1.45 (0.69–3.08)	14	1.41 (0.68–2.96)	1.35 (0.74–2.27)	1.41 (0.77–2.36)
*p*–trend		0.56		0.56	0.22	0.2
**Pneumonitis**						
Unexposed^d^	d.s.	1	d.s.	1	d.s.	d.s.
Exposed^e^	23	not shown	23	2.94 (0.97 – 8.89)	0.67 (0.43–1.01)	0.64 (0.41–0.97)
0–3.12	12	2.23 (0.69–7.27)	12	2.24 (0.69–7.26)	0.51 (0.27–0.89)	0.50 (0.26–0.87)
>3.12	11	4.73 (1.41–15.76)	11	4.65 (1.39–15.50)	1.03 (0.51–1.84)	0.94 (0.47–1.69)
*p*-trend		0.007		0.008	0.009	0.015

d.s. Data suppressed to comply with the NCI-UPitt data transfer agreement.

^a^ppm-years (lagged 10 years), NCI data taken from Koutros et al. (21) Table 3 and Web Table 4.

^b^HRs and RRs adjusted for race, sex, age, calendar time, and salary/wage classification.

^c^SMRs adjusted for race, sex, age, and calendar time.

^d^Baseline category for HRs.

^e^HRs are based on unlagged cumulative exposures (21).

**Table 3:** Observed deaths and UPitt external rate-based SMRs^a^ (regional rates) and 95% CIs for lung and bronchus cancer by cumulative acrylonitrile exposure category (lagged by 10 years) and study plant, full cohort, in the follow-up period 1942–2011.

**Cumulative exposure to acrylonitrile^b^**	**Study plant** ^**c**^							
	**1**	**2**	**3**	**4**	**5**	**6**	**7**	**8**
Unexposed	11 0.57 0.28–1.02	18 0.67 0.40–1.06	33 0.89 0.61–1.25	22 1.11 0.70–1.68	65 0.71 0.55–0.90	33 0.84 0.58–1.17	d.s.	73 0.66 0.52–0.83
Exposed	59 0.68 0.52–0.88	22 0.60 0.37–0.90	12 0.83 0.43–1.45	88 1.11 0.89–1.37	136 0.62 0.52–0.73	61 0.84 0.65–1.08	51 0.73 0.54–0.96	116 0.76 0.63–0.91
0–0.09	11 0.91 0.45–1.62	d.s.	d.s	26 1.24 0.81–1.81	29 0.75 0.50–1.07	17 0.71 0.41–1.14	d.s	18 0.63 0.37–1.00
>0.09–0.64	16 0.75 0.43–1.22	d.s	d.s	11 0.69 0.34–1.24	18 0.40 0.24–0.63	24 1.07 0.69–1.60	11 0.63 0.31–1.12	27 0.68 0.45–0.99
>0.64–2.30	13 0.61 0.33–1.04	d.s	d.s	10 0.96 0.46–1.76	22 0.61 0.38–0.92	13 0.74 0.39–1.26	11 0.68 0.34–1.21	33 0.77 0.53–1.08
>2.30–12.08	d.s.	d.s	d.s	12 0.73 0.38–1.27	27 0.59 0.39–0.86	d.s.	21 1.13 0.70–1.73	32 0.91 0.62–1.28
>12.08	11 0.93 0.46–1.66	d.s.	d.s.	29 1.88 1.26–2.70	40 0.74 0.53–1.01	d.s	d.s.	d.s.
*p*-trend	0.89	0.31	0.85	0.31	0.81	0.87	0.006	0.10

d.s Data suppressed to comply with NCI-UPitt data transfer agreement.

^a^SMRs adjusted for race, sex, age, and calendar time.

^b^ppm-years (lagged 10 years).

^c^Table cells show observed deaths, SMR, and 95% CI.

#### Misinformation about follow-up period for mesothelioma deaths

National Cancer Institute notes throughout their report that the risk period for mesothelioma mortality was limited to the years of the ICD-10 (1999–2011) as this was the first ICD revision to include specific codes for mesothelioma (ICD-10 code C45) (21). However, as shown in Table 1, our U.S. rate-based SMR analysis for mesothelioma replicated exactly the NCI SMR of 2.24 (95% CI = 1.39–3.42) for the full cohort only when we used the entire 1942–2011 observation period. When we limited the observation period to ICD-10, we found a slightly larger SMR for mesothelioma of 2.36 (95% CI = 1.46–3.61) (Table 1).

### External mortality comparisons

Table 1 shows a comparison of the NCI U.S. rate-based SMRs with UPitt SMRs based on U.S. and regional rates by AN exposure status for the four *a priori* cause of death categories. The numbers of deaths are identical for each cause of death category and with a few exceptions probably due to the aforementioned inconsistencies in follow-up periods used by NCI, and U.S. rate-based SMRs are close in value. For lung cancer and mesothelioma, UPitt regional rate-based SMRs are markedly less than U.S. rate-based SMRs reflecting higher rates of deaths for these two causes in the regional areas of the study plants. The UPitt U.S. rate-based SMR for mesothelioma among AN-exposed workers decreased from a statistically significant 2.48 (95% CI = 1.42–4.03) to a non-statistically significant < 2-fold risk (SMR = 1.70, 95% CI = 0.97–2.76). The UPitt U.S. and regional rate-based SMRs for bladder cancer and pneumonitis were nearly identical overall and by AN exposure status.

**Figure 1:**
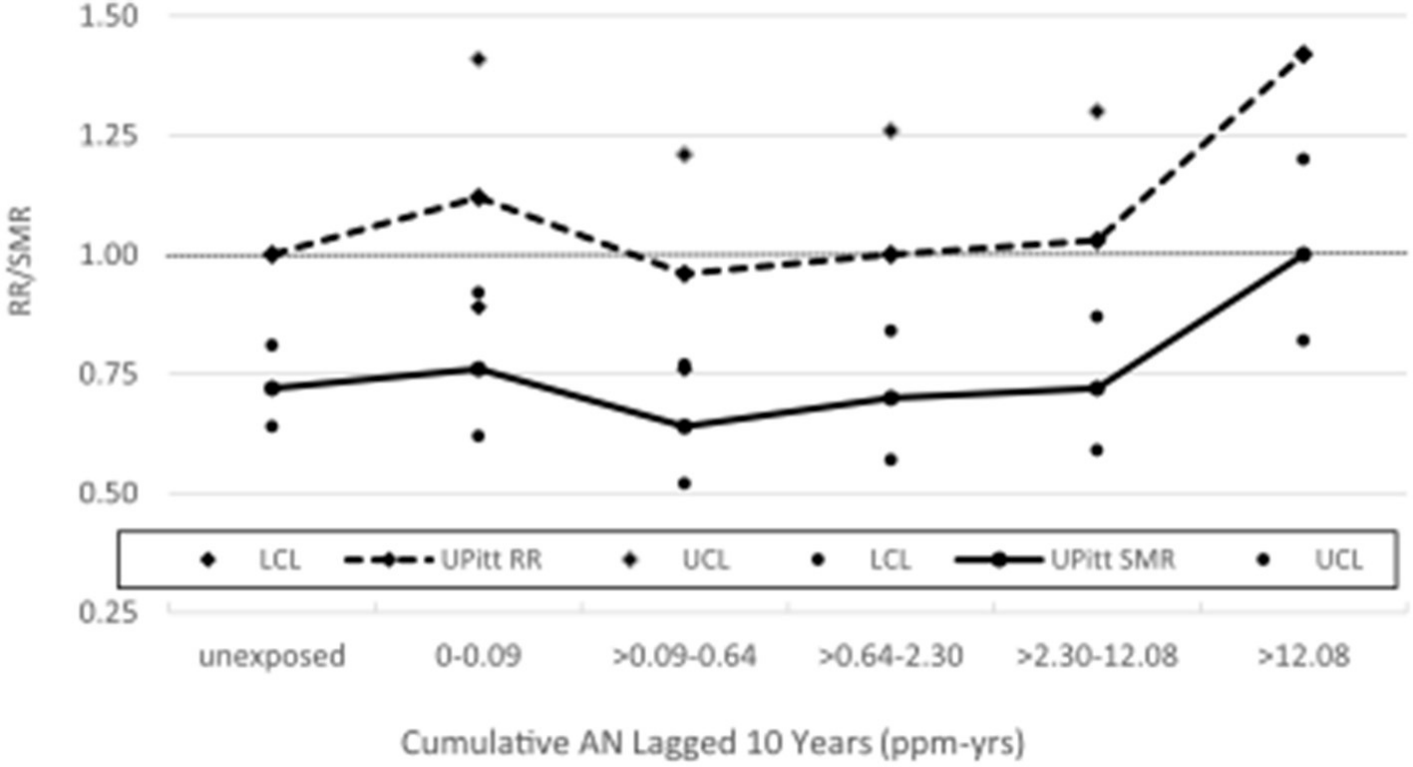
UPitt lung cancer relative risks (RR) and regional rate-based SMRs by cumulative AN exposure.

Table 2 shows a comparison of the NCI and UPitt observed counts, internal rate-based HRs (NCI) and RRs (UPitt), and UPitt U.S. and regional rate-based SMRs by NCI's cumulative AN exposure categories for the four *a priori* causes of death categories. Despite minor discrepancies in observed deaths for a few AN exposure categories, HRs and RRs were comparable for all causes of death and AN exposure categories. For lung cancer, regional rate-based SMRs were consistently less than U.S. rate-based SMRs, and with the exception of the highest AN exposure category, all represented statistically significant deficits in deaths. NCI's statistically significant 1.43-fold excess for the highest AN exposure category is fundamentally a result of comparing a null SMR of 1.00 to a statistically significant 28% deficit in deaths (SMR = 0.72, 95% CI = 0.64–0.81) for the unexposed baseline category (in fact, this actual ratio = 1.00 / 0.72 = 1.39). The UPitt regional rate-based SMRs also show less evidence of a linear trend with cumulative AN exposure than the NCI HRs (*p*-trend = 0.09 vs. 0.05, respectively). The UPitt SMRs and RRs and corresponding 95% CIs for lung cancer are graphed in Figure 1.

Regional rate-based SMRs for mesothelioma are less than those based on U.S. rates and show no statistically significant excesses in deaths across the unexposed and exposed AN categories (data suppressed for some categories) with no evidence of a linear trend with exposure (trend *p* = 0.72). Moreover, the UPitt U.S.-based SMR for mesothelioma in the highest cumulative AN exposure category (>1.33 ppm-years) decreased from a statistically significant to a nonstatistically significant < 2-fold risk based on regional rates (values not shown due to data suppression). U.S. and regional rate-based SMRs were similar for bladder cancer and revealed risk estimates, and AN exposure–response relationship trends were similar to the internal rate analyses. For pneumonitis, Table 2 shows a pattern of findings similar to those noted earlier for lung cancer. That is, the statistically significant 4.73-fold HR for workers in the highest cumulative AN exposure category is fundamentally due to a 6% deficit in deaths (regional SMR = 0.94, 95% CI = 0.47–1.69) being compared to an inordinately large, statistically significant deficit in deaths among the unexposed workers (actual ratio = 3.76; values not shown due to data suppression).

Table 3 shows plant-specific observed numbers of deaths, regional rate-based SMRs, and 95% CIs for lung cancer by AN cumulative exposure quintile. Corresponding internal RRs were discussed but not explicitly reported by Blair et al. (5) and Koutros et al. (21). While not shown here, the U.S. rate-based SMRs were higher than those based on regional rates for all plants except Plant 3, where the regional SMRs were only slightly larger. Among workers in the unexposed baseline category, Table 3 shows deficits in lung cancer deaths, ranging from a statistically significant SMR for Plant 7 to 0.89 (95% CI = 0.61–1.25) for Plant 3. Only unexposed workers in Plant 4 have an SMR slightly larger than expected (SMR = 1.11, 95% CI = 0.70–1.68).

For only one plant did we observe a slight lung cancer excess among the combined AN-exposed workers compared with the unexposed (Plant 4, SMR = 1.11, 95% CI = 0.89–1.37). We observed only one statistically significant excess in lung cancer deaths among the AN exposure categories examined (Plant 4, >12.08 ppm-years, SMR = 1.88, 95% CI = 1.26–2.70) but with no evidence of an exposure–response relationship (trend *p* = 0.31). With the exception of Plant 7, no plant revealed evidence of an AN exposure–response relationship. Trends in SMRs are difficult to discern in Table 3, due to the small number of observed deaths involved in many of the plant-specific exposure categories (including suppressed data). The statistically significant trend p for Plant 7 was apparently driven by the inordinately large statistically significant deficit of deaths among unexposed workers (value not shown due to data suppression).

#### Plant 4 sensitivity analysis

Our results in Tables 1, 2 indicate substantial variability in regional background mortality rates for mesothelioma. There are also some pieces of evidence in Table 3 that Plant 4 is responsible for a substantial portion of this variability. That is, Plant 4 was the only plant where AN-unexposed workers had an elevated risk of lung cancer and the only plant with a statistically significant excess in lung cancer deaths among the AN exposure categories examined.

### Indirect adjustment for potential confounding at the cohort level

#### Negative control outcome confounding adjustment

**Figure 2:**
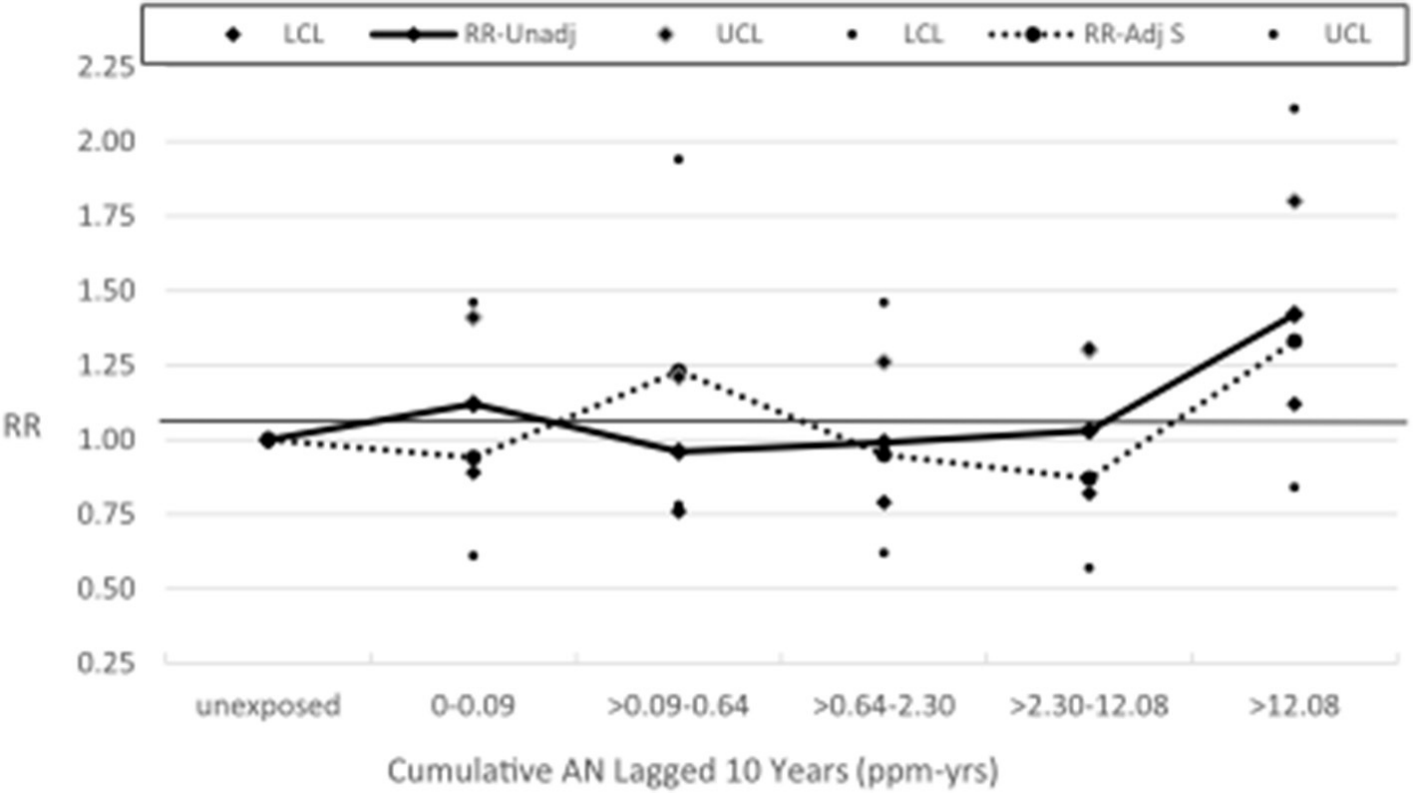
UPitt lung cancer relative risks (RR) by cumulative AN exposure, unadjusted and adjusted for confounding by smoking using the Richardson method.

Table 4 shows the results of our application of the Richardson method (28, 29) to indirectly adjust lung cancer RRs from the exposure–response relationship analysis for lung cancer presented in Table 2 for potential confounding by smoking and also shows observed deaths and RRs for lung cancer and COPD categorized into “unexposed” and “exposed” groups and by the NCI categories of the cumulative and average intensity of AN exposure (AIE). The unexposed categories served as the baseline category in the models in Table 4. Table 4 shows that the RRs for COPD for all AN-exposed workers and all but one cumulative AN exposure category were larger than 1.0 (ranging from 1.04 to 1.19), indicating a small degree of positive confounding by smoking, and the RR for the second cumulative AN exposure category was below 1.0 (0.78), indicating a moderate degree of negative confounding by smoking. Using these RRs for COPD to adjust for confounding by smoking, the unadjusted lung cancer RR for all workers exposed to AN and for all but the one cumulative exposure category reduced toward or below the null value. The RR for workers in the highest cumulative AN exposure category reduced from 1.43 to 1.33 and is no longer statistically significant (95% CI = 0.84–2.11); however, the Richardson confidence intervals tend to be conservative. Our adjusted RRs for lung cancer also provide much less evidence of a positive association with cumulative AN exposure (UPitt trend *p*-value = 0.63 vs. 0.07 for NCI). We note that our cohort-level adjusted RR for this highest exposure category is nearly identical to the smoking status-adjusted RR from NCI's case-cohort study (RR = 1.32, 95% CI = 0.55–3.17). A graph of the unadjusted and adjusted RRs and 95% CIs for lung cancer is shown in Figure 2. For AIE, three of the COPD RRs were below and two were above 1.0, indicating inconsistent evidence of confounding by smoking. The RR for the highest AIE, which showed evidence of positive confounding, reduced from 1.21 to 1.07.

**Table 4:** UPitt Lung and bronchus cancer relative risks (RR) in relation to AN exposure adjusted for potential confounding by smoking using the Richardson method, full cohort, 1942–2011.

	**Unadjusted lung and bronchus cancer**		**Chronic obstructive pulmonary disease (COPD)**		**Adjusted lung and bronchus cancer**
	**Obs**	**RR**^a^ **(95%) CI**	**Obs**	**RR**^a^ **(95%) CI**	**RR** ^a^ **(95%) CI**
Unexposed^b^	263	1.0	107	1.0	1.0
Exposed	545	1.08 (0.92–1.26)	215	1.05 (0.81–1.34)	1.03 (0.77–1.39)
**Cum AN exposure** ^c^					
0–0.09	109	1.12 (0.89–1.41)	42	1.19 (0.82–1.72)	0.94 (0.61–1.46)
>0.09–0.64	111	0.96 (0.76–1.21)	35	0.78 (0.53–1.16)	1.23 (0.78–1.94)
>0.64–2.30	107	1.00 (0.79–1.26)	47	1.04 (0.73–1.49)	0.95 (0.62–1.46)
>2.30–12.08	109	1.03 (0.82–1.30)	54	1.19 (0.85–1.68)	0.87 (0.57–1.31)
>12.08	109	1.42 (1.12–1.80)	37	1.06 (0.72–1.58)	1.33^e^ (0.84–2.11)
*p*-trend		0.07		0.57	0.63
**AIE AN Exposure** ^d^					
0–0.06	105	0.97 (0.77–1.23)	40	0.99 (0.68–1.45)	0.98 (0.63–1.53)
>0.06–0.14	109	1.20 (0.95–1.51)	47	1.26 (0.88–1.80)	0.95 (0.62–1.45)
>0.14–0.37	115	1.04 (0.83–1.30)	44	0.97 (0.68–1.41)	1.06 (0.69–1.64)
>0.37–1.46	107	1.03 (0.82–1.30)	40	0.92 (0.63–1.34)	1.11 (0.72–1.73)
>1.46	109	1.21 (0.96–1.53)	44	1.13 (0.78–1.64)	1.07 (0.69–1.67)
*p*-trend		0.48		0.54	0.59

^a^RRs adjusted for race, sex, age, calendar time, salary/wage classification, and plant.

^b^Baseline category for RRs.

^c^Cumulative AN exposure, ppm-years (lagged 10 years).

^d^Average intensity of AN exposure ppm (lagged 10 years).

^e^NCI ever-smoking adjusted RR based on a 10% sample with imputed values is 1.32 (0.55–3.17).

**Figure 3:**
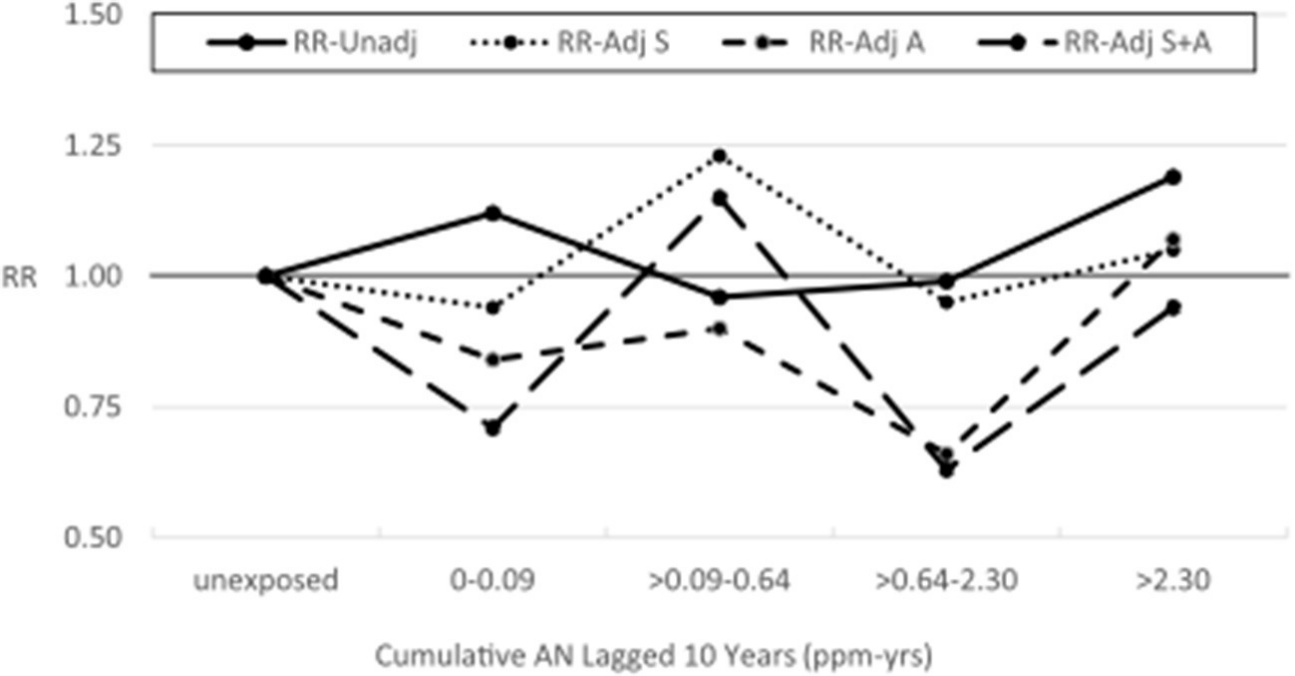
Lung cancer relative risks by cumulative AN exposure, full cohort—unadjusted and adjusted for smoking using the Richardson method (Continuous Data).

For lung cancer, we also fitted the two-parameter log-linear-quadratic model used by NCI to the continuous cumulative AN exposure (lagged 10 years) and applied the Richardson method to adjust this model for potential confounding by smoking (sparse data precluded modeling mesothelioma) using the corresponding parameter estimates for COPD. Figure 3, which is similar to Figure 1 in Koutros et al. (21) for the unadjusted model, shows that RRs from the adjusted model fall uniformly below those of the unadjusted model, corroborating our findings from the categorical analysis. Supplemental Table 8A provides details of the unadjusted and adjusted models. It should be noted that the linear and quadratic parameter estimates for lung cancer (and COPD) in both models (and COPD) are very close to 0 (even closer for the adjusted model) and neither is statistically significant, indicating no evidence of a positive AN exposure–response relationship as is evident in Figure 3.

Supplemental Tables 2–7 show plant-specific results for Plants 1 and 4–8 in the format of those presented in Table 4 for the combined cohort. Small numbers of lung cancer deaths precluded the analysis of Plants 2 and 3, and due to the small number of observed COPD deaths in some plants analyzed, many cell values were suppressed. In Plants 1, 4, 6, and 7, we observed evidence of positive confounding by smoking *via* the decreased adjusted RRs for many of the cumulative AN exposure categories examined. In Plant 6, adjustment for confounding led to a statistically significant inverse trend in RRs by cumulative AN exposure. In Plant 7, a statistically significant trend in unadjusted RRs was attenuated and no longer statistically significant based on the adjusted RRs. For AIE, in which we collapsed the five original exposure categories into two categories for Plants 6 and 7 and three for the remaining plants to minimize data suppression. We observed generally consistent evidence of positive confounding by smoking in Plants 4, 6, and 7, resulting in reduced adjusted RRs. Positive confounding was most pronounced in Plants 4 and 7 where adjusted RRs were markedly reduced, and in Plant 7, there was much less evidence of exposure–response relationship (unadjusted trend *p* = 0.01 vs. adjusted trend *p* = 0.19).

**Figure 4:**
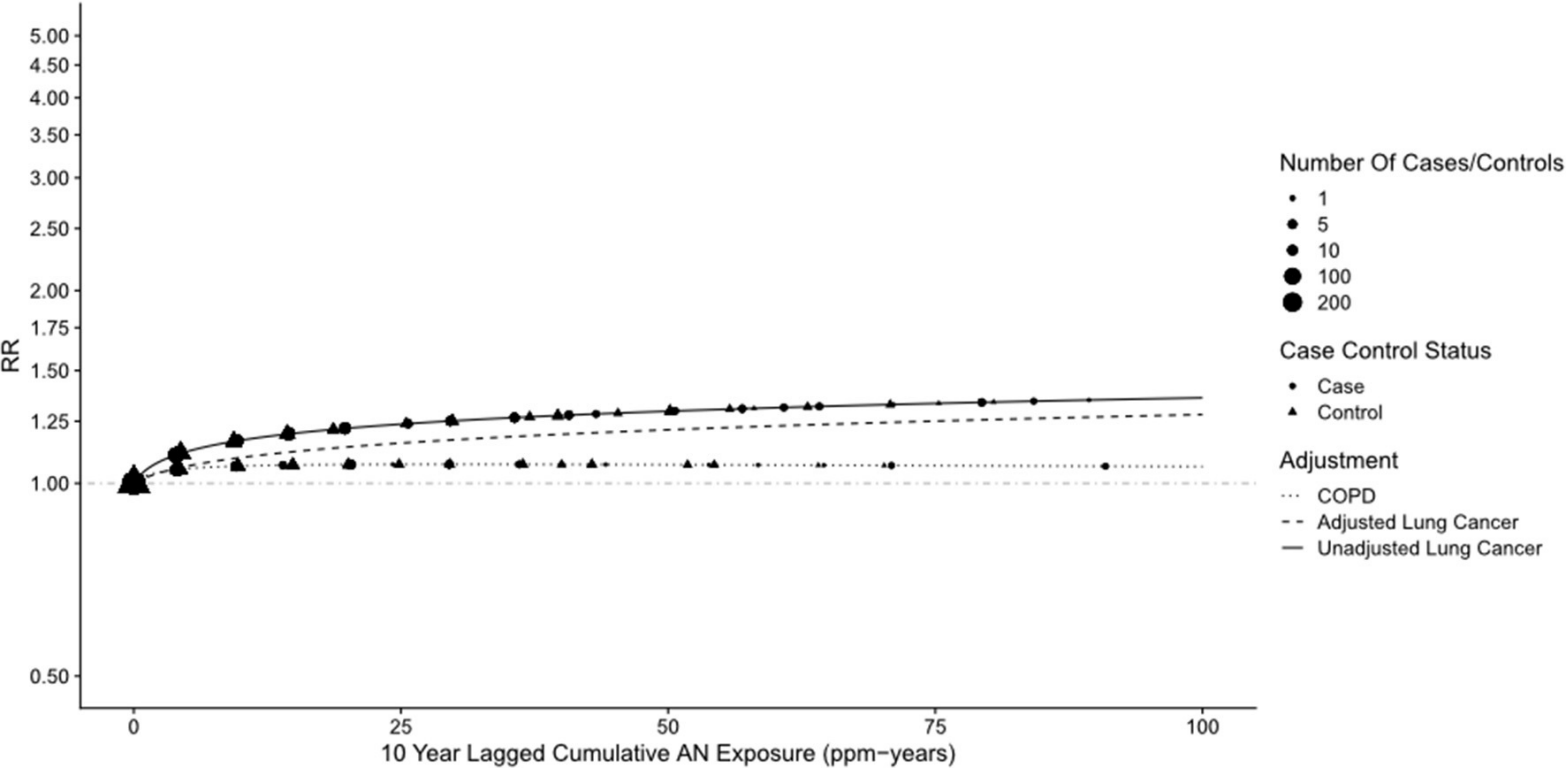
UPitt lung cancer relative risks (RR) by cumulative AN exposure, unadjusted and adjusted for confounding by smoking (S) and asbestos (A) using the Richardson method.

Table 5 shows the results of our application of the Richardson method to indirectly adjust lung cancer RRs for potential confounding by asbestos exposure. To minimize data suppression, we combined the last two cumulative AN exposure categories (now >2.30 ppm-years) and the last two AN AIE categories (now >0.37 ppm). For all AN-exposed workers and every cumulative AN exposure category, the adjusted RRs for lung cancer were reduced indicating a consistent, moderate level of positive confounding by asbestos exposure. In addition, as with the smoking adjustment, the adjusted RRs show much less evidence of an AN exposure–response relationship. Similarly, for AIE, three of four adjusted RRs were reduced showing less evidence of exposure–response relationship. Table 6 and Figure 4 show the results of adjusting lung cancer RRs for both smoking and for asbestos exposure using the same categories as Table 5. RRs for all but the second exposed category were further reduced with the RR for the highest cumulative AN exposure category now showing a deficit in risk (RR = 0.94, 95% CI = 0.26–3.34).

**Table 5:** UPitt lung and bronchus cancer relative risks (RR) in relation to AN exposure adjusted for potential confounding by asbestos using the Richardson method, full cohort, 1942–2011.

	**Unadjusted lung and bronchus cancer**		**Mesothelioma**		**Adjusted lung and bronchus cancer**
	**Obs**	**RR**^a^ **(95%) CI**	**Obs**	**RR**^a^ **(95%) CI**	**RR**^a^ **(95%) CI**
Unexposed^b^	263	1.0	d.s.	d.s.	1.0
Exposed	545	1.08 (0.92–1.26)	16	1.22 (0.43–3.44)	0.88 (0.31–2.52)
**Cum AN exposure** ^c^					
0–0.09	109	1.12 (0.89–1.41)	d.s.	d.s.	0.84 (0.19–3.70)
>0.09–0.64	111	0.96 (0.76–1.21)	d.s.	d.s.	0.90 (0.21–3.93)
>0.64–2.30	107	0.99 (0.79–1.25)	d.s.	d.s.	0.66 (0.17–2.54)
>2.30	218	1.19 (0.98–1.44)	d.s.	d.s.	1.07 (0.31–3.67)
*p*-trend		0.18		0.83	0.99
**AIE AN exposure** ^d^					
0–0.06	111	0.98 (0.78–1.23)	d.s.	d.s.	0.79 (0.18–3.47)
>0.06–0.14	105	1.18 (0.93–1.49)	d.s.	d.s.	0.51 (0.14–1.86)
>0.14–0.37	113	1.05 (0.83–1.32)	d.s.	d.s.	1.46 (0.27–7.82)
>0.37	216	1.11 (0.92–1.34)	d.s.	d.s.	1.06 (0.31–3.62)
*p*-trend		0.14		0.39	0.72

d.s. Data suppressed to comply with the NCI-UPitt data transfer agreement.

^a^RRs adjusted for race, sex, age, calendar time, and salary/wage classification.

^b^Baseline category for RRs.

^c^Cumulative AN exposure, ppm-years (lagged 10 years).

^d^Average intensity of AN exposure ppm (lagged 10 years).

**Table 6:** UPitt lung and bronchus cancer relative risks (RR) in relation to AN exposure adjusted for potential confounding by smoking and by asbestos using the Richardson method, full cohort, 1942–2011.

	**Unadjusted lung and bronchus cancer**		**Adjusted lung and bronchus cancer**
	**Obs**	**RR**^a^ **(95%) CI**	**RR**^a^ **(95%) CI**
Unexposed^b^	263	1.0	1.0
Exposed	545	1.08 (0.92–1.26)	0.84 (0.29–2.48)
**Cum AN exposure** ^c^			
0–0.09	109	1.12 (0.89–1.41)	0.71 (0.15–3.25)
>0.09–0.64	111	0.96 (0.76–1.21)	1.15 (0.25–5.29)
>0.64–2.30	107	0.99 (0.79–1.25)	0.63 (0.15–2.55)
>2.30	218	1.19 (0.98–1.44)	0.94 (0.26–3.34)
*p*-trend		0.18	0.89
**AIE AN exposure** ^d^			
0–0.06	111	0.98 (0.78–1.23)	0.80 (0.17–3.65)
>0.06–0.14	105	1.18 (0.93–1.49)	0.40 (0.11–1.53)
>0.14–0.37	113	1.05 (0.83–1.32)	1.53 (0.27–8.53)
>0.37	216	1.11 (0.92–1.34)	1.04 (0.29–3.7)
*p*-trend		0.14	0.74

^a^RRs adjusted for race, sex, age, calendar time, and salary/wage classification.

^b^Baseline category for RRs.

^c^Cumulative AN exposure, ppm-years (lagged 10 years).

^d^Average intensity of AN exposure ppm (lagged 10 years).

Table 7 shows the results of our application of the Richardson method to indirectly adjust bladder cancer RRs for potential confounding by smoking. For all AN-exposed workers and every NCI AN cumulative exposure category, the RRs for COPD were slightly larger than 1.0 (ranging from 1.01 to 1.07), indicating a small degree of positive confounding by smoking. This resulted in only slight reductions in the bladder cancer RRs and a similar lack of evidence for a positive exposure–response relationship. Similar findings were observed for AIE. Small numbers of observed deaths precluded plant-specific analyses of the data in Tables 5–7. Our application of the Richardson method to adjust pneumonitis RRs for potential confounding by smoking revealed only slight evidence of positive confounding and minor reductions in the RRs by cumulative AN exposure shown in Table 2 (data not shown).

**Table 7:** UPitt urinary bladder cancer relative risks (RR) in relation to AN exposure adjusted for potential confounding by smoking using the Richardson method, full cohort, 1942–2011.

	**Unadjusted urinary bladder cancer**		**Chronic obstructive pulmonary disease (COPD)**		**Adjusted urinary bladder cancer**
	**Obs**	**RR**^a^ **(95%) CI**	**Obs**	**RR**^a^ **(95%) CI**	**RR** ^a^ **(95%) CI**
Unexposed^b^	16	1.0	107	1.0	1.0
Exposed	39	0.91 (0.50–1.65)	215	1.05 (0.81–1.34)	0.87 (0.45–1.66)
**Cum AN exposure** ^c^					
0–0.37	12	0.80 (0.37–1.72)	70	1.04 (0.76–1.43)	0.76 (0.33–1.75)
>0.37-6.69	13	0.73 (0.35–1.55)	96	1.07 (0.80–1.43)	0.69 (0.31–1.53)
>6.69	14	1.41 (0.67–2.96)	49	1.01 (0.70–1.44)	1.40 (0.62–3.19)
*p*-trend		0.55		0.85	0.64
**AIE AN exposure** ^d^					
0–0.26	12	0.52 (0.24–1.13)	113	1.06 (0.80–1.40)	0.50 (0.22–1.12)
>0.26–2.56	12	0.82 (0.38–1.76)	74	0.99 (0.73–1.36)	0.83 (0.36–1.89)
>2.56	15	3.20 (1.53–6.72)	28	1.19 (0.77–1.84)	2.70 (1.14–6.37)
*p*-trend		0.01		0.67	0.03

^a^RRs adjusted for race, sex, age, calendar time, and salary/wage classification.

^b^Baseline category for RRs.

^c^Cumulative AN exposure, ppm-years (lagged 10 years).

^d^Average intensity of AN exposure ppm (lagged 10 years).

#### Plant 4 sensitivity analysis for lung cancer

**Figure 5:**
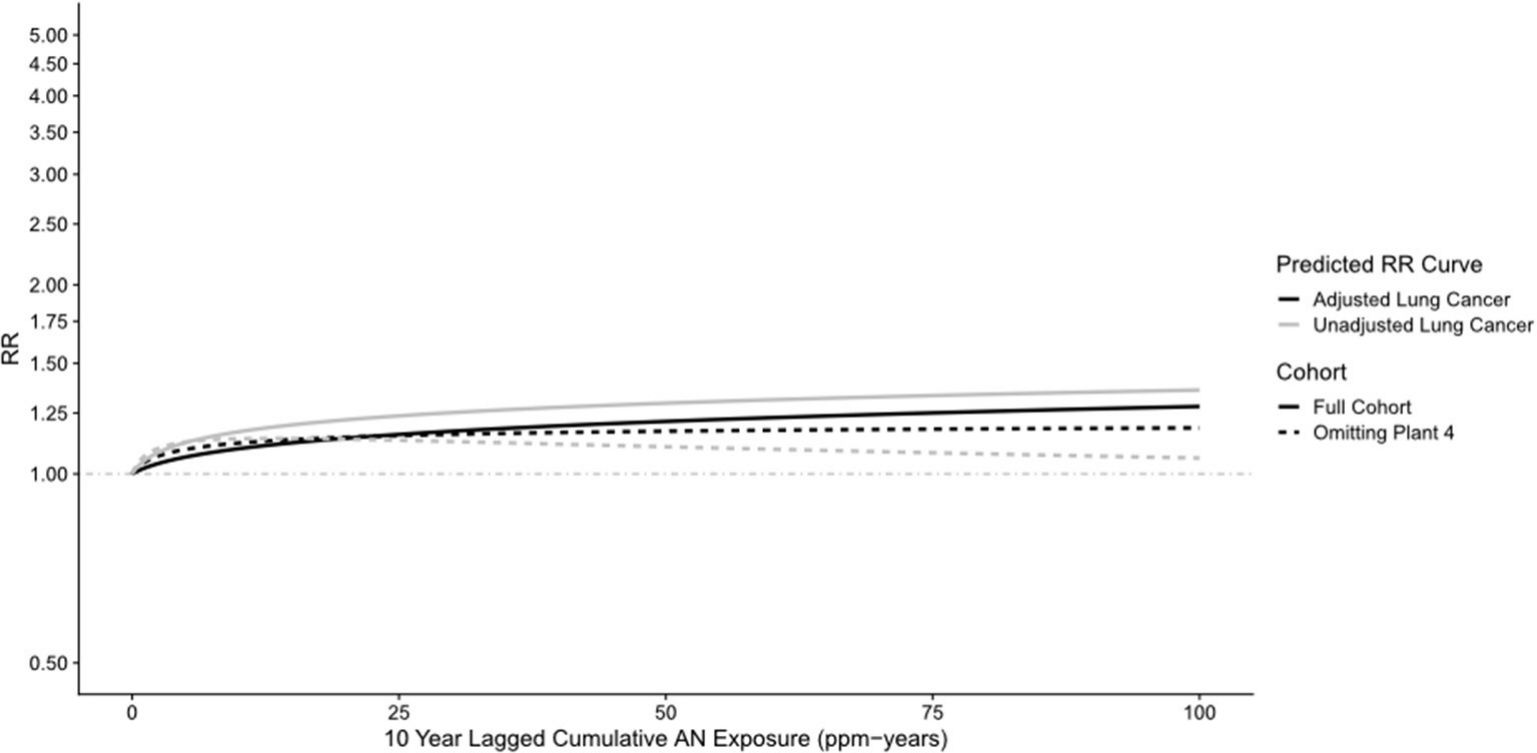
UPitt lung cancer relative risk: full cohort vs. omitting plant 4—unadjusted and adjusted for smoking using the Richardson method (continuous data).

**Figure 6:**
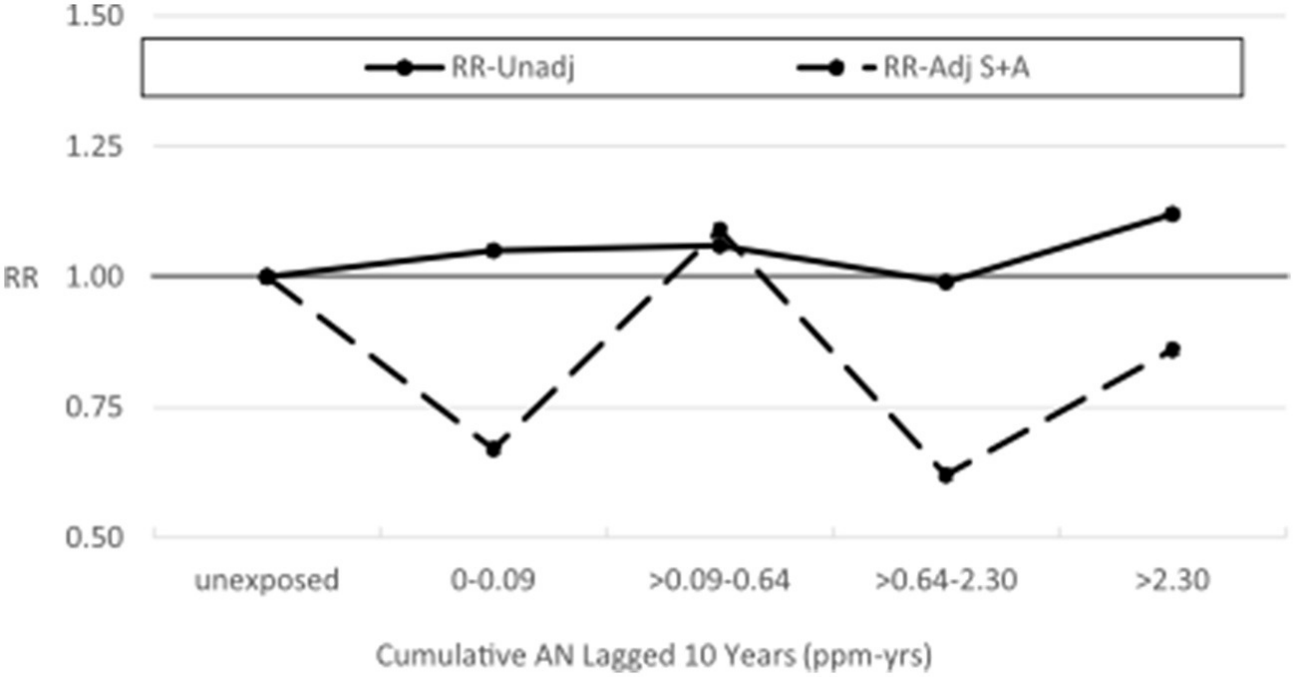
UPitt lung cancer relative risks (RR) by cumulative AN exposure, unadjusted and adjusted for confounding by smoking (S) and by asbestos (A) using the Richardson method, omitting Plant 4.

Supplemental Tables 8B, 9–11 show results similar to Supplementary Tables 4–6, 8A but with Plant 4 omitted. In Supplemental Table 9, the unadjusted RR for lung cancer in the highest cumulative AN category (>12.08 ppm-years) was reduced from a statistically significant 1.42 (95% CI: 1.12–1.80) to a non-statistically significant 1.23 (95% CI: 0.94–1.60), and the trend *p*-value increased from 0.07 to 0.30. A similar pattern of findings was also observed for the highest category of the average intensity of AN exposure (>1.46 ppm). Similarly, in the log-linear-quadratic model (Supplementary Table 8B), the coefficients are closer to 0 indicating less evidence of an exposure–response relationship with cumulative AN exposure. This attenuated association is also evident in Figure 5 which compares the log-linear quadratic models for the full cohort and full cohort omitting Plant 4. The omission of Plant 4 had a similar effect of reducing lung cancer RRs and evidence of exposure–response relationship in models adjusted for asbestos (Table 5 vs. Supplementary Table 10) and for smoking and asbestos (Table 6 vs. Supplementary Table 11). Figure 6 compares the unadjusted lung cancer RRs to RRs adjusted for both smoking and asbestos for the full cohort omitting Plant 4 (Supplementary Table 11). In analyses adjusted for asbestos with Plant 4 omitted, sparse data required collapsing the two highest cumulative AN exposure categories into one (> 2.30 ppm-years) and precluded the comparison of the corresponding continuous data models.

#### Bias adjustment factors and Monte Carlo sensitivity analysis

Table 8 shows the results of our cohort-level indirect adjustment of lung and bladder cancer SMRs for potential confounding by smoking. The ever-smoking prevalence among workers from all plants is considerably greater than the corresponding rates in the standard state populations, indicating that unadjusted SMRs were heavily and positively confounded by smoking. This led to lung cancer CRRs that exceeded 2.0 for all but two plants (data not shown due to suppression) and a CRR of 1.61 for bladder cancer. Thus, lung and bladder cancer unadjusted SMRs were reduced to less than null values in all cases and all but one deficit in deaths was statistically significant. Notably, the statistically significant unadjusted lung cancer SMR for Plant 4 (SMR = 1.88, 95% CI = 1.26–2.70) was reduced to a statistically significant 34% deficit in deaths (SMR = 0.66, 95% CI = 0.46–0.95).

**Table 8:** Confounding risk ratios (CRR) and smoking-adjusted SMRs for lung and bladder cancer based on estimated cohort smoking prevalence rates, showing unadjusted SMRS by plant and overall, full cohort, 1942–2011.

**Plant/state**	**Estimated point prevalence of ever smoking**		**UPitt unadjusted SMR 95% CI (Highest cumulative AN category)**	**Confounding risk ratio (CRR)^d^**	**Smoking- adjusted SMR (Highest cum AN Cat)**	**Monte Carlo based 95% confidence interval on adjusted SMR**
	**Cohort AN exposed** ^a, b^	**Corresponding state** ^c^				
**Lung cancer**						
1 FL	0.71	0.249	0.93 (0.46–1.66)	2.52	0.37	0.21–0.66
2 TX	0.53	0.271	d.s.	d.s.	d.s.	d.s.
3 OH	0.62	0.316	d.s.	d.s.	d.s.	d.s.
4 VA	0.78	0.237	1.88 (1.26–2.70)	2.85	0.66	0.46–0.95
5 AL	0.71	0.300	0.74 (0.53–1.01)	2.16	0.34	0.25–0.47
6 LA	0.68	0.263	d.s.	d.s.	d.s.	d.s.
7 IN	0.67	0.285	d.s.	d.s.	d.s.	d.s.
8 TX	0.65	0.271	d.s.	d.s.	d.s.	d.s.
All plants	0.68	0.271^e^	1.00 (0.82–1.20)	2.25	0.44	0.37–0.53
**Bladder cancer** ^f^						
All plants	0.68	0.271^e^	1.41 (0.77–2.36)	1.61	0.62	0.52–1.46

^a^Estimated for the full cohort by NCI from interview data obtained *via* nested case-cohort study (5).

^b^Because NCI did not obtain smoking histories for lung cancer cases identified in the update (but imputed values instead) or provide the imputed values or plant-specific smoking prevalence rates (21), plant-specific smoking prevalence was estimated by identifying the proportion of plant-specific smokers and multiplying by 0.68 / 0.63 = 1.08, where 0.68 is reported ever-smoking prevalence for a full cohort based on NCI imputed values and 0.63 is the ever-smoking prevalence available on NCI data file.

^c^Obtained from Behavioral Risk Factor Surveillance System Survey, 1995, data for men (42).

^d^Based on the “all plants” smoking prevalence rate *via* the case–cohort control study (5) and the relative risk of smoking and lung cancer of 19.1 (21).

^e^Median value of prevalence rates for states shown in Table.

^f^Based on the relative risk of smoking and bladder cancer of 3.5 (41).

## Discussion

Our evaluation of the relationship between AN exposure and lung cancer mortality using external comparisons based on plant-specific regional mortality rates produced results similar to those that we reported in our reanalysis of the first NCI AN cohort study follow-up (25). We observed statistically significant deficits in lung cancer deaths among unexposed workers in contrast to SMRs at or near the null value among the most highly exposed workers. This indicates that the statistically significant 1.43-fold lung cancer excess among workers in the highest quintile of cumulative AN exposure (lagged 10 years) reported by NCI using internal rate comparisons (HRs) was fundamentally a result of comparing a null value to a statistically significant deficit (21). In this second reanalysis, we observed a similar but more pronounced pattern for pneumonitis mortality in which a statistically significant 4.73-fold excess stemmed from comparing a 6% deficit in deaths among the highest exposure to a large, statistically significant deficit among the unexposed.

As we discussed in our first reanalysis (25), several possible explanations exist for the large differences in lung cancer relative risks in this study population when internal or external comparison rates are used. First, internal comparisons produce more valid results because selection bias stemming from the healthy worker effect can reduce the putative effect of high exposure to acrylonitrile when external comparison rates are used. However, the NCI cohort has now been followed for several decades and much of any healthy worker effect present in the first update has attenuated. In addition, the selection for workers who are healthy at the time of hire is usually more relevant for chronic cardiovascular and non-malignant respiratory diseases than lung cancer, which has a relatively sudden onset, short survival time, and high case-fatality rate (24). Second, external comparisons produce more valid results because the unexposed group has a different underlying lung cancer risk than the exposed group. The inordinately low SMRs for lung cancer among unexposed workers overall are puzzling given that we used regional standard population rates. As regional rates can help adjust for the social, cultural, and economic factors related to diseases such as lung cancer and even help to adjust for geographic variability in tobacco use, it is difficult to postulate what non-occupational factors may have had such a profound influence on the lung cancer mortality experience of the unexposed workers. Supplemental Table 12 compares the distribution of the major demographic factors of unexposed and exposed workers in the NCI cohort. Factors possibly related to AN exposure (e.g., sex, age at hire, year of hire, and wage class) do not differ markedly between unexposed and exposed workers, and our adjustments for these factors in internal relative risk models for lung cancer likely did not lead to any residual confounding.

Third, given the large number of lung cancer deaths and the overall robustness of the NCI study, the chance or under-ascertainment of deaths are unlikely explanations for the low SMRs among unexposed workers. Finally, the possibility remains that some heretofore unknown selection factors for low lung cancer incidence were operating on members of this cohort or that some type of protective effect for lung cancer arose from a particular exposure or combination of exposures encountered at the study plants. Some of these explanations also apply to our findings for pneumonitis although the heterogeneous nature of this cause of death category and their uncertain etiologies further complicate their interpretation. Consequently, the underlying reason for the inordinately low SMRs among unexposed workers remains unknown.

In the NCI study, investigators have attempted to adjust lung cancer risk estimates for confounding by smoking *via* a nested case-cohort study of lung cancer based on a 10% random sample of the cohort. Blair et al. (5) initially reported an exceedingly low RR for lung cancer among ever smokers compared to never smokers of 3.6 (95% CI = 1.6–8.2), suggesting that smoking status was most likely misclassified among subjects. This observation was confirmed later by Cunningham in 2005 (26). Blair et al.'s finding contrasts starkly with the corresponding HR of 19.1 (95% CI = 5.3–68.9) reported by Koutros et al. (21) in the updated cohort based on imputed smoking data for the new lung cancer cases. While the more recent estimate is in line with well-known relative risks for smoking and lung cancer, it is not clear why this estimate increased so dramatically from the first update and was reported with much less precision, especially considering that 646 new lung cancer cases that were added to the case-cohort study in the recent update. Koutros et al. (21) also limited smoking adjustment to lung cancer HRs among workers in the highest cumulative AN exposure category, thus precluding an evaluation of how smoking adjustment impacted AN exposure–response relationships.

In contrast, our application of the Richardson indirect method enabled adjustment of lung cancer risks for potential confounding by smoking at the cohort level and for workers in all categories of AN exposure. In addition, unlike the NCI study, this method enabled additional adjustment of lung cancer risks for asbestos exposure and smoking adjustment of bladder cancer risks. Consequently, our cohort-level adjustment of lung cancer RRs for confounding by smoking and asbestos yielded for the total cohort, and within the eight study plants (smoking adjustment only), mostly had decreased RRs and much less evidence of a positive association with cumulative AN exposure than reported by NCI.

In the current reanalysis, we recognized markedly lower mesothelioma SMRs based on regional vs. U.S. death rates as well as uniquely higher lung cancer SMRs among unexposed and exposed workers from Plant 4 and noted that the local area includes the Newport News Shipyards where many asbestos-containing materials were used historically. Shipyard work is associated with elevated lung cancer and mesothelioma risks, and some Plant 4 workers (and persons in the local general population) may have been employed for some time in the yards. Despite the elevated lung cancer rates among Plant 4 workers, we observed no mesothelioma deaths among Plant 4 workers (21 mesothelioma deaths occurred in Plants 1, 3, 5, 6, 7, and 8).

While our Richardson lung cancer adjustment for asbestos likely accounted for at least some of any shipyard-related asbestos exposures that may have occurred among Plant 4 workers, residual confounding from asbestos exposures unique to Plant 4 may remain in the cohort. Thus, we repeated key mortality comparisons omitting or isolating workers from Plant 4. This sensitivity analysis of the remaining cohort revealed decreased lung cancer mortality risks and even less evidence of an AN exposure–response relationship, particularly in RR models adjusted for both smoking and asbestos exposure. Thus, while Plant 4 comprised only 13.3% of the total cohort, it had a relatively large impact on the overall findings for lung cancer that were not recognized in the NCI study (5, 21).

As expected from the small relative risk for smoking and bladder cancer, our application of the Richardson method to bladder cancer revealed little evidence of positive confounding by smoking, and a similar lack of a positive relationship with cumulative AN exposure as reported by NCI (21). In an independent cohort study of Plant 3 workers, (14) a statistically significant excess risk for bladder cancer based on four observed deaths was reported. An expanded Plant 3 study to investigate the bladder cancer excess (15) and a further expansion and extended follow-up of the cohort (16) found that the bladder cancer excess decreased to a non-statistically significant level. As noted by Koutros et al., because of high survival rates, bladder cancer risks are best evaluated in studies that include both incidence cases and deaths.

Our cohort-level adjustments for smoking based on internal mortality comparisons were corroborated by our smoking adjustment of regional rate-based SMRs for lung and bladder cancer among workers in the highest cumulative AN exposure category. This analysis revealed considerably higher smoking rates among workers compared with plant-specific standard state populations indicating that unadjusted lung and bladder cancer SMRs in the NCI study were heavily and positively confounded by smoking.

The results of our reanalysis of the 2011 update of the NCI study continue to reflect the lack of clear and consistent evidence of an association between AN exposure and mortality from lung cancer both across earlier studies and within the current NCI study. In the latter case, we observed considerable inconsistencies in results when using external vs. internal mortality comparisons and when considering potential confounding by smoking and/or asbestos and/or the impact of Plant 4. Given that consistent evidence of elevated risks and exposure–response relationships across and within studies are requisites to establish a causal association, the absence of such overall evidence argues against a causal association between AN and lung cancer.

## Conclusion

Overall, our reanalysis provided little evidence to support NCI's suggestion of associations between AN exposure and mortality from lung and bladder cancer and pneumonitis. NCI's conclusions were driven by exposure–response relationships stemming from exceedingly low baseline rates, lack of cohort-level adjustment for confounding by smoking and/or asbestos or within the categories of AN exposure reported by the NCI study authors, and no accounting for the potential impact of historical shipyard-related asbestos exposures in the local area of Plant 4.

## Data availability statement

The data analyzed in this study is subject to the following licenses/restrictions: The data that support the findings of this study are available from the National Cancer Institute but restrictions apply to the availability of these data, which were used for the current study under a data transfer agreement between the NCI and the UPitt, and so are not publicly available. Requests to access these datasets should be directed to Stella Koutros, KoutrosS@mail.nih.gov.

## Ethics statement

The studies involving human participants were reviewed and approved by Institutional Review Board of the University of Pittsburgh. Written informed consent for participation was not required for this study in accordance with the national legislation and the institutional requirements.

## Author contributions

GM designed and directed the statistical analysis used in the reanalysis of the NCI acrylonitrile cohort data and played a major role in the preparation of the manuscript. AK conducted and contributed to the design of the statistical analysis and contributed to the preparation of the manuscript. Both authors contributed to the article and approved the submitted version.

## Footnotes

^1^0.68 is the reported ever-smoking prevalence for full cohort based on NCI imputed values (21) and 0.63 is the ever-smoking prevalence available to us on NCI data file.
