# Supplementary material for: A reevaluation of selected mortality risks in the updated NCI/NIOSH acrylonitrile cohort study
Source: Front Public Health. 2023 Apr 6;11:1122346. doi: 10.3389/fpubh.2023.1122346 (PMC10117843; doi:10.3389/fpubh.2023.1122346)
Supplement: Supplementary file 1 [file Data_Sheet_1.zip › Supplementary Material/Data Sheet 1.DOCX]

**Appendix A**

**Details of Reformatting and Validating the NCI Cohort Data File**

***Data Imputation and Reformatting:*** OCMAP-Plus (1998) requires that key demographic and work history dates are defined in detail as month/day/year. The NCI cohort file did not include certain key dates such as date as death requiring us to impute these dates as functions of other provided dates as described in detail below. Also, all dates included in the NCI cohort file specified year only. Thus, it was necessary to randomly assign month and day. Details of our date imputation process are as follows:

Demographic and General Work History Variables

**Date of Birth**: Only the year of birth was present in the NCI file, named DOBYR. Date of birth was imputed using this DOBYR variable and a randomly generated month and day.

**Date of Death**: The NCI file provided no date information on date of death. For subjects coded as deceased in the NCI file, we added the value of the NCI variable named Age_at_Exit_Decimal to the DOBYR variable to calculate the date of death year, DODYR. We imputed date of death using this DODYR variable and a randomly generated month and day.

**Hire Date**: The NCI data only contains the hire year, it is named HIREYR. We imputed hire date (HIRE_DT) using HIREYR and a randomly generated month and day.

**Job Start**: The beginning of the first job. Set to HIRE_DT.

**Job Stop**: The end date of the last job on the work history. We assumed that work histories ended on December 31^st^ of the year specified by the NCI variable ENDYR.

Detailed Work Histories

OCMAP requires that work histories be expressed as individual job entries (i.e., for each job, start date, stop date, job code and average AN exposure level must be specified). Because the NCI work history and AN exposure data were provided in a highly summarized form (work histories expressed as annual estimates of time exposed and cumulative AN exposure level) reformatting was necessary. This conversion required two key assumptions about the calendar time distribution of the summary work history data, which was not specified on the NCI cohort file:

1 For exposure time that did not span a full year, the exposed portion of the annual work history occurred at the beginning of the year. The remainder of the annual work history was filled with a dummy non-exposed job.

2 Because only annual estimates of exposure and number of days exposed per year were available, individual jobs did not span more than one full year.

Specifically, for each worker, individual work histories were recreated using from the NCI cohort file date of hire, date of termination, stop date for person-years, date of first exposed job and end date of last exposed job. To meet the specifications of OCMAP-Plus, the job start date of the n^th^ job on the work history was set to the job stop date of the (n-1)^th^ job. The process of reconstruction was as follows.

If the first job was not exposed, then:

1. The job start date of the first job on the work history was set to the date of hire.
2. The job stop date of the first job was set to the date of first exposure (if the date of hire was

equal to the date of first exposure, then the date of first exposure became the job start date of the first job on the work history).

1. The job start date of the second job on the work history was set to the date of first exposure.
2. The job stop date of the second job was set to the date of first exposure plus the number of days exposed during that year of exposure. Because only start date of first exposure and end date of last exposure were available from the NCI cohort file, jobs with partial years of exposure were given a start date of January 1 of that exposure year. The associated job stop date was set to January 1 of that year plus the number of days exposed in that year. The remainder of that exposed year was filled with a dummy non-exposed job. If there were no days exposed in a given year, then the associated job was given a start date of January 1 of that year and a stop date of January 1 of the following year. Subsequent jobs in the work history were created in a similar manner.
3. The job stop date of the last job on the work history was set to the date of termination (if the date of termination was equal to the end date of last exposure, then the stop date of the last job was set to the end date of last exposure).
4. If the first job was exposed then start at Step 3 and treat as the first job.

To satisfy OCMAP-Plus job exposure formatting requirements, we computed daily average AN exposure estimates by dividing the NCI annual cumulative AN exposure estimates by the number of days exposed in the corresponding year. The NCI cohort file contained AN exposure estimates accumulated across individual years beginning in 1942 and ending in 1983, along with the associated number of days exposed during those individual years. The documentation of the NCI cohort file noted that the cumulative AN exposure estimates were weighted by a factor of 5/7, presumably to adjust for time off work.

***Validation:*** To validate the reformatting process prior to our statistical analysis, we attempted to replicate selected results of the Koutros et al. (2019) analysis. The Appendix Table shows the absolute and relative differences in person counts between the NCI and our cohort file for selected study variables for the full cohort and for deaths in the four *a priori* cause of death categories. For the full cohort, the number of subjects was overall and by plant were identical, and the total person-years differed by only 2,774 or 0.27%. The number of subjects unexposed and exposed to AN was identical for each of the four *a priori* cause of death categories and the number of subjects in the quintile categories of cumulative exposure to AN differed only slightly (by one or two deaths) in only two of five categories for two of the cause of death categories considered (lung and bronchus cancer and urinary bladder cancer). Well not shown here, only negligible differences were also noted for other study variables considered including duration of exposure to AN and average exposure to AN. We considered the differences in counts noted between the two cohort files to be negligible and unimportant for our reevaluation of cause of death-specific mortality risks.

**Appendix Table**

**Comparability of NCI and UPitt AN cohort files, full cohort and deaths in *a priori***

**cause of death categories, follow-up period 1942-2011**

| **Study Factor** | **NCI**  **(Koutros et al. 2019)** | **UPitt** | **Absolute difference^a.^** | **Relative difference^b.^** |
| --- | --- | --- | --- | --- |
| **Full Cohort ^c.^** | | | | |
| **Total Subjects**  **Total Person-Years** | 25,460  1,023,922 | 25,460  1,026,696 | 0  2774 | 0%  0.27% |
| **Plant number**  1  2  3  4  5  6  7  8 | 1,896  1,989  1,545  3,379  7,321  2,653  2,339  4,338 | 1,896  1,989  1,545  3,379  7,321  2,653  2,339  4,338 | 0  0  0  0  0  0  0  0 | 0%  0%  0%  0%  0%  0%  0%  0% |
| **AN exposure**  Unexposed  Exposed | 8,571  16,889 | 8,571  16,889 | 0  0 | 0%  0% |
| **Lung and Bronchus Cancer Deaths ^d.^** | | | | |
| **AN exposure**  Unexposed  Exposed  Total | 249  559  808 | 249  559  808 | 0  0  0 | 0%  0%  0% |
| **Cumulative exposure lagged 10-years (ppm-yrs)**  0-0.09  >0.09-0.64  >0.64-2.30  >2.30-12.08  >12.08 | 109  109  109  109  109 | 109  111  107  109  109 | 0  2  -2  0  0 | 0%  1.83%  -1.83%  0%  0% |
| **Urinary Bladder Cancer Deaths ^d.^** | | | | |
| **AN exposure**  Unexposed  Exposed  Total | 16  39  55 | 16  39  55 | 0  0  0 | 0%  0%  0% |

| **Cumulative exposure lagged 10 years (ppm-yrs)**  0-0.37  >0.37-6.69  >6.69 | 13  13  13 | 12  13  14 | -1  0  1 | 7.69%  0%  -7.69% |
| --- | --- | --- | --- | --- |
| **Deaths due to Pneumonitis from Solids and Liquids ^d.^** | | | | |
| **AN exposure**  Unexposed  Exposed  Total | d.s.  23  27 | d.s.  23  27 | 0  0  0 | 0%  0%  0% |
| **Cumulative exposure lagged 10 years (ppm-yrs)**  0-3.12  >3.12 | 12  11 | 12  11 | 0  0 | 0%  0% |
| **Mesothelioma Deaths (ICD10 only 1999-2011) ^d.^** | | | | |
| **AN exposure**  Unexposed  Exposed  Total | d.s.  16  21 | d.s.  16  21 | 0  0  0 | 0%  0%  0% |
| **Cumulative exposure lagged 10 years (ppm-yrs)**  0-1.33  >1.33 | d.s.  d.s. | d.s.  d.s. | 0  0 | 0%  0% |

d.s. data suppressed to comply with NCI-UPitt data transfer agreement

a. UPitt file minus NCI

b. (UPitt file minus NCI) / NCI

c. Categories and counts reported by Koutros et al. (2019) Table 1

d. Categories and counts reported by Koutros et al. (2019) Table 3 and Web Table 4
