# Supplementary material for: A reevaluation of selected mortality risks in the updated NCI/NIOSH acrylonitrile cohort study
Source: Front Public Health. 2023 Apr 6;11:1122346. doi: 10.3389/fpubh.2023.1122346 (PMC10117843; doi:10.3389/fpubh.2023.1122346)
Supplement: Supplementary file 1 [file Data_Sheet_1.zip › Supplementary Material/Data Sheet 2.DOCX]

**Appendix B**

Based on the Richardson’s method (2010, 2014), the estimated adjusted RR for lung cancer can be calculated as the exponentiated difference between the log estimated RR for lung cancer and log estimated RR for the negative control disease for each confounder (either COPD for smoking or mesothelioma for asbestos) (Equation 1):

$${\hat{\boldsymbol{RR}}}_{\boldsymbol{adjusted}}\boldsymbol{=}\exp\left\{ \boldsymbol{Log}\left( {\hat{\boldsymbol{RR}}}_{\boldsymbol{lung cancer}} \right)\boldsymbol{-Log}\left( {\hat{\boldsymbol{RR}}}_{\boldsymbol{ischemic}} \right) \right\}$$

**=** $\exp\left\{ {\hat{\boldsymbol{\beta}}}_{\boldsymbol{lung cancer}}\boldsymbol{-}{\hat{\boldsymbol{\beta}}}_{\boldsymbol{control}} \right\}$ **, (1)**

where $\hat{\boldsymbol{\beta}}\boldsymbol{=Log}\left( \hat{\boldsymbol{RR}} \right)\boldsymbol{.}$

Thus, the estimated variance of $\hat{\boldsymbol{\beta}}$ can be calculated using the Delta method (Equation 2):

$\hat{\boldsymbol{\beta}}\boldsymbol{=}\hat{\boldsymbol{Var}}\left( {\hat{\boldsymbol{\beta}}}_{\boldsymbol{lung cancer}} \right)\boldsymbol{+}\hat{\boldsymbol{Var}}\left( {\hat{\boldsymbol{\beta}}}_{\boldsymbol{control}} \right)\boldsymbol{- 2*}\hat{\boldsymbol{Cov}}\left( {\hat{\boldsymbol{\beta}}}_{\boldsymbol{lung cancer}}\boldsymbol{,}{\hat{\boldsymbol{\beta}}}_{\boldsymbol{control}} \right)$ **(2)**

We calculated the variance estimate for ${\hat{\boldsymbol{\beta}}}_{\boldsymbol{lung cancer}}\boldsymbol{-}{\hat{\boldsymbol{\beta}}}_{\boldsymbol{ischemic}}$ approximately as the sum of the two variance estimates for ${\hat{\boldsymbol{\beta}}}_{\boldsymbol{lung cancer}}$ and ${\hat{\boldsymbol{\beta}}}_{\boldsymbol{ischemic}}$ without considering the covariance between the beta estimates (which can only be estimated in the joint modeling of the two outcomes). In general this covariance can likely be expected to be positive due to joint effect of the underlying confounder, which results in the estimated variance being artificially large and the corresponding confidence intervals conservative. Using the adjusted variance, we calculated the conservative 95% confidence interval for the adjusted RR as:

$\boldsymbol{exp}\left( {\hat{\boldsymbol{\beta}}}_{\boldsymbol{adjusted}}\boldsymbol{\pm1.96*}\sqrt{{\hat{\boldsymbol{Var}}\boldsymbol{(RR}}_{\boldsymbol{adjusted}}\boldsymbol{)}} \right)$ **(3)**
