# Supplementary material for: A reevaluation of selected mortality risks in the updated NCI/NIOSH acrylonitrile cohort study
Source: Front Public Health. 2023 Apr 6;11:1122346. doi: 10.3389/fpubh.2023.1122346 (PMC10117843; doi:10.3389/fpubh.2023.1122346)
Supplement: Supplementary file 1 [file Data_Sheet_1.zip › Supplementary Material/Table 1.DOCX]

**Supplemental Table 1**

**Observed deaths, NCI U.S. rate-based SMRs^a.,b.^, UPitt U.S. and regional rate-based SMRs^b.^ for**

**key causes of death, by AN exposure, full NCI AN cohort, 1942-2011 (2012)**

|  | **All causes of death** | | **Lung and bronchus** | | **Urinary bladder**  **(underlying cause only)** | |
| --- | --- | --- | --- | --- | --- | --- |
|  | **Obs** | **SMR (95%CI)** | **Obs** | **SMR (95%CI)** | **Obs** | **SMR (95%CI)** |
| **Total Cohort** | | | | | | |
| **NCI (U.S.) 2011** | 8124 | 0.79 (0.78-0.81) | 808 | 0.87 (0.81-0.93) | 55 | 0.84 (0.63-1.10) |
| **UPitt (regional) 2011** | 8124 | 0.73 (0.72-0.75) | 808 | 0.74 (0.69-0.79) | 55 | 0.81 (0.61-1.05) |
| **UPitt (regional) 2012** | 8124 | 0.70 (0.68-0.71) | 808 | 0.71 (0.66-0.76) | 55 | 0.76 (0.57-0.99) |
| **AN-Unexposed** | | | | | | |
| **NCI (U.S.) 2011** | 2731 | 0.82 (0.79-0.85) | 249 | 0.84 (0.74-0.95) | 16 | 0.81 (0.46-1.31) |
| **UPitt (regional) 2011** | 2731 | 0.75 (0.72-0.78) | 249 | 0.73 (0.64-0.82) | 16 | 0.77 (0.44-1.25) |
| **UPitt (regional) 2012** | 2731 | 0.71 (0.69-0.74) | 249 | 0.70 (0.62-0.79) | 16 | 0.72 (0.41-1.18) |
| **AN-Exposed** | | | | | | |
| **NCI (U.S.) 2011** | 5393 | 0.79 (0.77-0.81) | 559 | 0.88 (0.81-0.96) | 39 | 0.86 (0.61-1.17) |
| **UPitt (regional) 2011** | 5393 | 0.72 (0.71-0.74) | 559 | 0.74 (0.68-0.80) | 39 | 0.83 (0.59-1.13) |
| **UPitt (regional) 2012** | 5393 | 0.69 (0.67-0.71) | 559 | 0.71 (0.66-0.77) | 39 | 0.77 (0.55-1.06) |

1. From Koutros et al. (2019) Table 2
2. SMRs adjusted for race, sex, age and calendar time
