# Supplementary material for: A reevaluation of selected mortality risks in the updated NCI/NIOSH acrylonitrile cohort study
Source: Front Public Health. 2023 Apr 6;11:1122346. doi: 10.3389/fpubh.2023.1122346 (PMC10117843; doi:10.3389/fpubh.2023.1122346)
Supplement: Supplementary file 1 [file Data_Sheet_1.zip › Supplementary Material/Table 10.DOCX]

**Supplemental Table 9**

**UPitt Lung and Bronchus Cancer Relative Risks (RR) in Relation to AN Exposure Adjusted for Potential Confounding by Smoking Using Richardson’s Method, Full Cohort Omitting Plant 4, 1942-2011**

|  | **Unadjusted Lung and**  **Bronchus Cancer** | | **Chronic Obstructive Pulmonary Disease (COPD)** | | **Adjusted Lung and Bronchus Cancer** |
| --- | --- | --- | --- | --- | --- |
|  | **Obs** | **RR^a.^ (95%) CI** | **Obs** | **RR^a.^ (95%) CI** | **RR ^a.^ (95%) CI** |
| **Unexposed^b.^** | 241 | 1.0 | 103 | 1.0 | 1.0 |
| **Exposed** | 457 | 1.05 (0.89 –1.25) | 175 | 0.97 (0.74 – 1.27) | 1.08 (0.79 – 1.48) |
| **Cum AN Exposure^c.^** |  |  |  |  |  |
| 0-0.09 | 83 | 1.06 (0.82 – 1.38) | 31 | 1.04 (0.69 – 1.58) | 1.02 (0.62 – 1.66) |
| >0.09-0.64 | 100 | 1.00 (0.78 – 1.27) | 32 | 0.8 (0.53 – 1.20) | 1.25 (0.78 – 2.01) |
| >0.64-2.30 | 97 | 0.99 (0.78 – 1.27) | 41 | 0.99 (0.67 – 1.44) | 1.01 (0.64 – 1.58) |
| >2.30-12.08 | 97 | 1.05 (0.82 – 1.35) | 44 | 1.12 (0.77 – 1.62) | 0.94 (0.61 – 1.48) |
| >12.08 | 80 | 1.23 (0.94 –1.60) | 27 | 0.93 (0.6 – 1.46) | 1.32^e.^ (0.78 – 2.21) |
| p-trend |  | 0.30 |  | 0.96 | 0.60 |
| **AIE AN Exposure^d.^** |  |  |  |  |  |
| 0-0.06 | 91 | 0.97 (0.76 – 1.25) | 34 | 0.93 (0.62 – 1.39) | 1.05 (0.65 – 1.69) |
| >0.06-0.14 | 108 | 1.25 (0.99 – 1.58) | 46 | 1.27 (0.88 – 1.82) | 0.99 (0.64 – 1.52) |
| >0.14-0.37 | 88 | 0.98 (0.76 – 1.26) | 31 | 0.83 (0.54 – 1.26) | 1.19 (0.73 – 1.94) |
| >0.37-1.46 | 95 | 1.02 (0.80 – 1.31) | 33 | 0.84 (0.56 – 1.26) | 1.22 (0.76 – 1.95) |
| >1.46 | 75 | 1.04 (0.80– 1.37) | 31 | 1.01 (0.66 – 1.55) | 1.03 (0.62 – 1.71) |
| p-trend |  | 0.76 |  | 0.60 | 0.59 |

1. RRs adjusted for race, sex, age, calendar time, salary/wage classification and plant
2. Baseline category for RRs
3. Cumulative AN exposure, ppm-years (lagged 10 years)
4. Average intensity of AN exposure ppm (lagged 10 years)
5. NCI ever smoking adjusted RR based on 10% sample with imputed values is 1.32 (0.55-3.17)
