# Supplementary material for: A reevaluation of selected mortality risks in the updated NCI/NIOSH acrylonitrile cohort study
Source: Front Public Health. 2023 Apr 6;11:1122346. doi: 10.3389/fpubh.2023.1122346 (PMC10117843; doi:10.3389/fpubh.2023.1122346)
Supplement: Supplementary file 1 [file Data_Sheet_1.zip › Supplementary Material/Table 11.DOCX]

**Supplemental Table 10**

**UPitt Lung and Bronchus Cancer Relative Risks (RR) in Relation to AN Exposure Adjusted for Potential Confounding by Asbestos Using Richardson’s Method, Full Cohort (omitting Plant 4), 1942-2011**

|  | **Unadjusted Lung and**  **Bronchus Cancer** | | **Mesothelioma** | | **Adjusted Lung and Bronchus Cancer** |
| --- | --- | --- | --- | --- | --- |
|  | **Obs** | **RR^a.^ (95%) CI** | **Obs** | **RR^a.^ (95%) CI** | **RR ^a.^ (95%) CI** |
| **Unexposed^b.^** | 241 | 1.0 | d.s. | 1.0 | 1.0 |
| **Exposed** | 457 | 1.05 (0.89–1.25) | 16 | 1.35 (0.48 3.82) | 0.78 (0.27–2.23) |
| **Cum AN Exposure^c.^** |  |  |  |  |  |
| 0-0.09 | 97 | 1.06 (0.82–1.38) | d.s. | d.s. | 0.70(0.16–3.12) |
| >0.09-0.64 | 104 | 1.00 (0.78–1.27) | d.s. | d.s. | 0.87 (0.20–3.78) |
| >0.64-2.30 | 86 | 0.99 (0.78–1.27) | d.s. | d.s. | 0.62 (0.16–2.42) |
| >2.30 | 170 | 1.12 (0.91–1.38) | d.s. | d.s. | 0.89 (0.26–3.08) |
| p-trend |  | 0.40 |  | 0.92 | 0.82 |
| **AIE AN Exposure^d.^** |  |  |  |  |  |
| 0-0.06 | 97 | 0.98 (0.77–1.26) | d.s. | d.s. | 0.77 (0.18–3.41) |
| >0.06-0.14 | 104 | 1.23 (0.97–1.57) | d.s. | d.s. | 0.54 (0.15–1.96) |
| >0.14-0.37 | 86 | 0.99 (0.77–1.28) | d.s. | d.s. | 1.17 (0.22–6.29) |
| >0.37 | 170 | 1.03 (0.84–1.27) | d.s. | d.s. | 0.85 (0.25–2.93) |
| p-trend |  | 0.74 |  | 0.90 | 0.95 |

d.s. Data suppressed to comply with NCI-UPitt data transfer agreement

1. RRs adjusted for race, sex, age, calendar time, salary/wage classification and plant
2. Baseline category for RRs
3. Cumulative AN exposure, ppm-years (lagged 10 years)
4. Average intensity of AN exposure ppm (lagged 10 years)
