# Supplementary material for: A reevaluation of selected mortality risks in the updated NCI/NIOSH acrylonitrile cohort study
Source: Front Public Health. 2023 Apr 6;11:1122346. doi: 10.3389/fpubh.2023.1122346 (PMC10117843; doi:10.3389/fpubh.2023.1122346)
Supplement: Supplementary file 1 [file Data_Sheet_1.zip › Supplementary Material/Table 12.DOCX]

**Supplemental Table 11**

**UPitt Lung and Bronchus Cancer Relative Risks (RR) in Relation to AN Exposure Adjusted for Potential Confounding by Smoking and by Asbestos Using Richardson’s Method, Full Cohort (omitting Plant 4), 1942-2011**

|  | **Unadjusted Lung and**  **Bronchus Cancer** | | **Adjusted Lung and Bronchus Cancer** |
| --- | --- | --- | --- |
|  | **Obs** | **RR^a.^ (95%) CI** | **RR ^a.^ (95%) CI** |
| **Unexposed^b.^** | 241 | 1.0 | 1.0 |
| **Exposed** | 457 | 1.05 (0.89–1.25) | 0.80 (0.27 2.38) |
| **Cum AN Exposure^c.^** |  |  |  |
| 0-0.09 | 83 | 1.06 (0.82–1.38) | 0.67 (0.14–3.16) |
| >0.09-0.64 | 100 | 1.00 (0.78–1.27) | 1.09 (0.23–5.02) |
| >0.64-2.30 | 97 | 0.99 (0.78–1.27) | 0.62 (0.15–2.58) |
| >2.30 | 177 | 1.12 (0.91–1.38) | 0.86 (0.24–3.09) |
| p-trend |  | 0.40 | 0.81 |
| **AIE AN Exposure^d.^** |  |  |  |
| 0-0.06 | 97 | 0.98 (0.77–1.26) | 0.83 (0.18–3.85) |
| >0.06-0.14 | 104 | 1.23 (0.97–1.57) | 0.42 (0.11–1.6) |
| >0.14-0.37 | 86 | 0.99 (0.77–1.28) | 1.46 (0.26–8.33) |
| >0.37 | 170 | 1.03 (0.84–1.27) | 0.94 (0.26–3.36) |
| p-trend |  | 0.74 | 0.95 |

1. RRs adjusted for race, sex, age, calendar time, salary/wage classification and plant
2. Baseline category for RRs
3. Cumulative AN exposure, ppm-years (lagged 10 years)
4. Average intensity of AN exposure ppm (lagged 10 years)
