# Supplementary material for: A reevaluation of selected mortality risks in the updated NCI/NIOSH acrylonitrile cohort study
Source: Front Public Health. 2023 Apr 6;11:1122346. doi: 10.3389/fpubh.2023.1122346 (PMC10117843; doi:10.3389/fpubh.2023.1122346)
Supplement: Supplementary file 1 [file Data_Sheet_1.zip › Supplementary Material/Table 13.DOCX]

**Supplemental Table 12**

**Demographic Characteristics of NCI AN Cohort by AN Exposure Status**

| **Variable** | **Unexposed** | | **Exposed** | | **Total** | |
| --- | --- | --- | --- | --- | --- | --- |
|  | **No.** | **%** | **No.** | **%** | **No.** | **%** |
| Race  White  Nonwhite | 7701  875 | 89.8  10.2 | 14671  2213 | 86.9  13.1 | 22372  3088 | 87.9  12.1 |
| Sex  Male  Female | 5255  3321 | 61.3  38.7 | 15015  1869 | 88.9  11.1 | 20270  5190 | 79.6  20.4 |
| Race/Sex  White Male  Nonwhite Male  White Female  Nonwhite Female | 4731  524  2970  351 | 55.2  6.1  34.6  4.1 | 13348  1667  1323  546 | 79.1  9.9  7.8  3.2 | 18079  2191  4293  897 | 71.0  8.6  16.9  3.5 |
| Age at Hire  15-24  25-34  35-44  45-54  55-64  65+ | 4165  2871  1166  319  49  6 | 48.6  33.5  13.6  3.7  0.6  0.1 | 8952  5409  2071  407  45  0 | 53.0  32.0  12.3  2.4  0.3  0 | 13117  8280  3237  726  94  6 | 51.5  32.5  72.6  2.9  0.4  0.0 |
| Wage Class  Salary  Wage | 2129  6447 | 24.8  75.2 | 4108  12776 | 24.3  75.7 | 6237  19223 | 24.5  75.5 |
| Wage Class/Sex  Salary Male  Salary Female  Wage Male  Wage Female | 1825  304  3430  3017 | 21.3  3.5  40.0  35.2 | 3916  192  11099  1677 | 23.2  1.1  65.7  9.9 | 5741  496  14529  4694 | 22.5  1.9  57.1  18.4 |
| Year of Hire  1942-49  1950-59  1960-69  1970-84 | 8  2459  2769  3340 | 0.1  28.7  32.3  38.9 | 14  4191  6729  5950 | 0.1  24.8  39.9  35.2 | 22  6650  9498  9290 | 0.1  26.1  37.3  36.5 |
