# Supplementary material for: A reevaluation of selected mortality risks in the updated NCI/NIOSH acrylonitrile cohort study
Source: Front Public Health. 2023 Apr 6;11:1122346. doi: 10.3389/fpubh.2023.1122346 (PMC10117843; doi:10.3389/fpubh.2023.1122346)
Supplement: Supplementary file 1 [file Data_Sheet_1.zip › Supplementary Material/Table 2.DOCX]

**Supplemental Table 2**

**UPitt Lung and Bronchus Cancer Relative Risks (RR) in Relation to AN Exposure Adjusted for Potential Confounding by Smoking Using Richardson’s Method, Plant 1, 1942-2011**

|  | **Unadjusted Lung and**  **Bronchus Cancer** | | **Chronic Obstructive Pulmonary Disease (COPD)** | | **Adjusted Lung and Bronchus Cancer** |
| --- | --- | --- | --- | --- | --- |
|  | **Obs** | **RR^a.^ (95%) CI** | **Obs** | **RR^a.^ (95%) CI** | **RR ^a.^ (95%) CI** |
| **Unexposed^b.^** | 11 | 1.0 | d.s. | 1.0 | 1.0 |
| **Exposed** | 59 | 0.94 (0.48–1.84) | 37 | 1.01 (0.41–2.51) | 0.93 (0.30–2.87) |
| **Cum AN Exposure^c.^** |  |  |  |  |  |
| 0-0.09 | 11 | 1.25 (0.53–2.95) | d.s. | d.s. | 0.91 (0.23–3.63) |
| >0.09-0.64 | 16 | 1.06 (0.48–2.30) | d.s. | d.s. | 1.79 (0.43–7.48) |
| >0.64-2.30 | 13 | 0.83 (0.36–1.92) | 10 | 1.23 (0.42–3.61) | 0.67 (0.17–2.62) |
| >2.30-12.08 | d.s. | d.s. | d.s. | d.s. | 0.60 (0.13–2.68) |
| >12.08 | 11 | 1.15 (0.47–2.80) | d.s. | d.s. | 0.97 (0.22–4.30) |
| p-trend |  | 0.48 |  | 0.93 | 0.62 |
| **AIE AN Exposure^d.^** |  |  |  |  |  |
| 0-0.37 | 33 | 1.02 (0.50–2.06) | 21 | 1.03 (0.4–2.63) | 0.99 (0.31–3.19) |
| >0.135-1.46 | 15 | 0.69 (0.31–1.55) | 10 | 0.91 (0.31–2.69) | 0.76 (0.20–2.93) |
| >1.46 | 11 | 1.42 (0.58–3.50) | d.s. | d.s. | 1.25 (0.27–5.66) |
| p-trend |  | 0.91 |  | 0.94 | 0.99 |

1. RRs adjusted for race, sex, age, calendar time, salary/wage classification
2. Baseline category for RRs
3. Cumulative AN exposure, ppm-years (lagged 10 years)
4. Average intensity of AN exposure ppm (lagged 10 years)
