# Supplementary material for: A reevaluation of selected mortality risks in the updated NCI/NIOSH acrylonitrile cohort study
Source: Front Public Health. 2023 Apr 6;11:1122346. doi: 10.3389/fpubh.2023.1122346 (PMC10117843; doi:10.3389/fpubh.2023.1122346)
Supplement: Supplementary file 1 [file Data_Sheet_1.zip › Supplementary Material/Table 3.DOCX]

**Supplemental Table 3**

**UPitt Lung and Bronchus Cancer Relative Risks (RR) in Relation to AN Exposure Adjusted for Potential Confounding by Smoking Using Richardson’s Method, Plant 4, 1942-2011**

|  | **Unadjusted Lung and**  **Bronchus Cancer** | | **Chronic Obstructive Pulmonary Disease (COPD)** | | **Adjusted Lung and Bronchus Cancer** |
| --- | --- | --- | --- | --- | --- |
|  | **Obs** | **RR^a.^ (95%) CI** | **Obs** | **RR^a.^ (95%) CI** | **RR ^a.^ (95%) CI** |
| **Unexposed^b.^** | 22 | 1.0 | d.s. | 1.0 | 1.0 |
| **Exposed** | 88 | 0.94 (0.56–1.57) | 40 | 1.41 (0.46–4.36) | 0.67 (0.19–2.31) |
| **Cum AN Exposure^c.^** |  |  |  |  |  |
| 0-0.09 | 26 | 1.11 (0.6–2.09) | 11 | 1.71 (0.49–5.92) | 0.65 (0.16–2.62) |
| >0.09-0.64 | 11 | 0.61 (0.29–1.3) | d.s. | d.s. | 1.03 (0.18–5.82) |
| >0.64-2.30 | 10 | 0.83 (0.38–1.8) | d.s. | d.s. | 0.50 (0.11–2.33) |
| >2.30-12.08 | 12 | 0.63 (0.3–1.31) | 10 | 1.69 (0.49–5.83) | 0.37 (0.09–1.57) |
| >12.08 | 29 | 1.71 (0.92–3.18) | 10 | 1.41 (0.38–5.2) | 1.21 (0.29–5.12) |
| p-trend |  | 0.37 |  | 0.64 | 0.93 |
| **AIE AN Exposure^d.^** |  |  |  |  |  |
| 0-0.37 | 15 | 0.76 (0.38–1.51) | d.s. | d.s. | 0.51 (0.12–2.21) |
| >0.135-1.46 | 39 | 0.87 (0.49–1.53) | 20 | 1.51 (0.47–4.84) | 0.57 (0.16–2.1) |
| >1.46 | 34 | 1.30 (0.71–2.37) | 13 | 1.13 (0.32–3.96) | 1.14 (0.28–4.60) |
| p-trend |  | 0.33 |  | 0.92 | 0.56 |

d.s. Data suppressed to comply with NCI-UPitt data transfer agreement

1. RRs adjusted for race, sex, age, calendar time, salary/wage classification
2. Baseline category for RRs
3. Cumulative AN exposure, ppm-years (lagged 10 years)
4. Average intensity of AN exposure ppm (lagged 10 years)
