# Supplementary material for: A reevaluation of selected mortality risks in the updated NCI/NIOSH acrylonitrile cohort study
Source: Front Public Health. 2023 Apr 6;11:1122346. doi: 10.3389/fpubh.2023.1122346 (PMC10117843; doi:10.3389/fpubh.2023.1122346)
Supplement: Supplementary file 1 [file Data_Sheet_1.zip › Supplementary Material/Table 4.DOCX]

**Supplemental Table 4**

**UPitt Lung and Bronchus Cancer Relative Risks (RR) in Relation to AN Exposure Adjusted for Potential Confounding by Smoking Using Richardson’s Method, Plant 5, 1942-2011**

|  | **Unadjusted Lung and**  **Bronchus Cancer** | | **Chronic Obstructive Pulmonary Disease (COPD)** | | **Adjusted Lung and Bronchus Cancer** |
| --- | --- | --- | --- | --- | --- |
|  | **Obs** | **RR^a.^ (95%) CI** | **Obs** | **RR^a.^ (95%) CI** | **RR ^a.^ (95%) CI** |
| **Unexposed^b.^** | 65 | 1.0 | 25 | 1.0 | 1.0 |
| **Exposed** | 136 | 0.82 (0.59–1.15) | 47 | 0.82 (0.46–1.44) | 1.01 (0.52–1.94) |
| **Cum AN Exposure^c.^** |  |  |  |  |  |
| 0-0.09 | 29 | 0.94 (0.59–1.52) | d.s. | d.s. | 1.34 (0.47–3.85) |
| >0.09-0.64 | 18 | 0.55 (0.32–0.95) | d.s. | d.s. | 0.76 (0.28–2.05) |
| >0.64-2.30 | 22 | 0.84 (0.50–1.39) | d.s. | d.s. | 0.97 (0.37–2.59) |
| >2.30-12.08 | 27 | 0.83 (0.52–1.34) | 14 | 1.14 (0.55–2.33) | 0.73 (0.31–1.73) |
| >12.08 | 40 | 0.96 (0.62–1.48) | 11 | 0.65 (0.29–1.43) | 1.48 (0.60–3.64) |
| p-trend |  | 0.74 |  | 0.66 | 0.84 |
| **AIE AN Exposure^d.^** |  |  |  |  |  |
| 0-0.37 | 54 | 0.94 (0.63–1.4) | 15 | 0.88 (0.44–1.79) | 1.06 (0.47–2.38) |
| >0.135-1.46 | 45 | 0.75 (0.5–1.13) | 14 | 0.64 (0.32–1.30) | 1.18 (0.52–2.65) |
| >1.46 | 37 | 0.77 (0.49–1.20) | 18 | 1.03 (0.51–2.09) | 0.75 (0.32–1.72) |
| p-trend |  | 0.14 |  | 0.79 | 0.59 |

1. RRs adjusted for race, sex, age, calendar time, salary/wage classification
2. Baseline category for RRs
3. Cumulative AN exposure, ppm-years (lagged 10 years)
4. Average intensity of AN exposure ppm (lagged 10 years)
