# Supplementary material for: A reevaluation of selected mortality risks in the updated NCI/NIOSH acrylonitrile cohort study
Source: Front Public Health. 2023 Apr 6;11:1122346. doi: 10.3389/fpubh.2023.1122346 (PMC10117843; doi:10.3389/fpubh.2023.1122346)
Supplement: Supplementary file 1 [file Data_Sheet_1.zip › Supplementary Material/Table 5.DOCX]

**Supplemental Table 5**

**UPitt Lung and Bronchus Cancer Relative Risks (RR) in Relation to AN Exposure Adjusted for Potential Confounding by Smoking Using Richardson’s Method, Plant 6, 1942-2011**

|  | **Unadjusted Lung and**  **Bronchus Cancer** | | **Chronic Obstructive Pulmonary Disease (COPD)** | | **Adjusted Lung and Bronchus Cancer** |
| --- | --- | --- | --- | --- | --- |
|  | **Obs** | **RR^a.^ (95%) CI** | **Obs** | **RR^a.^ (95%) CI** | **RR ^a.^ (95%) CI** |
| **Unexposed^b.^** | 33 | 1.0 | d.s. | 1.0 | 1.0 |
| **Exposed** | 61 | 0.87 (0.56–1.36) | 21 | 1.25 (0.54–2.93) | 0.69 (0.27–1.81) |
| **Cum AN Exposure^c.^** |  |  |  |  |  |
| 0-0.09 | 17 | 0.83 (0.45–1.52) | d.s. | d.s. | 1.91 (0.35–10.45) |
| >0.09-0.64 | 24 | 1.20 (0.70–2.08) | d.s. | d.s. | 2.03 (0.47–8.76) |
| >0.64-2.30 | 13 | 0.66 (0.34–1.28) | d.s. | d.s. | 0.55 (0.15–1.97) |
| >2.30-12.08 | d.s. | d.s. | d.s. | d.s. | 0.21 (0.05–0.96) |
| >12.08 | d.s. | d.s. | d.s. | d.s. | d.s. |
| p-trend |  | 0.36 |  | 0.00 | 0.00 |
| **AIE AN Exposure^d.^** |  |  |  |  |  |
| 0-0.37 | 37 | 0.97 (0.59–1.61) | 11 | 1.27 (0.49–3.31) | 0.77 (0.26–2.26) |
| >0.135 | 24 | 0.75 (0.43–1.31) | 10 | 1.24 (0.47–3.29) | 0.61 (0.20–1.87) |
| p-trend |  | 0.33 |  | 0.67 | 0.39 |

d.s. Data suppressed to comply with NCI-UPitt data transfer agreement

1. RRs adjusted for race, sex, age, calendar time, salary/wage classification
2. Baseline category for RRs
3. Cumulative AN exposure, ppm-years (lagged 10 years)
4. Average intensity of AN exposure ppm (lagged 10 years)
