# Supplementary material for: A reevaluation of selected mortality risks in the updated NCI/NIOSH acrylonitrile cohort study
Source: Front Public Health. 2023 Apr 6;11:1122346. doi: 10.3389/fpubh.2023.1122346 (PMC10117843; doi:10.3389/fpubh.2023.1122346)
Supplement: Supplementary file 1 [file Data_Sheet_1.zip › Supplementary Material/Table 6.DOCX]

**Supplemental Table 6**

**UPitt Lung and Bronchus Cancer Relative Risks (RR) in Relation to AN Exposure Adjusted for Potential Confounding by Smoking Using Richardson’s Method, Plant 7, 1942-2011**

|  | **Unadjusted Lung and**  **Bronchus Cancer** | | **Chronic Obstructive Pulmonary Disease (COPD)** | | **Adjusted Lung and Bronchus Cancer** |
| --- | --- | --- | --- | --- | --- |
|  | **Obs** | **RR^a.^ (95%) CI** | **Obs** | **RR^a.^ (95%) CI** | **RR ^a.^ (95%) CI** |
| **Unexposed^b.^** | d.s. | 1.0 | d.s. | d.s. | 1.0 |
| **Exposed** | 51 | 2.39 (0.97–5.90) | 13 | 1.70 (0.34–8.50) | 1.41 (0.22–8.95) |
| **Cum AN Exposure^c.^** |  |  |  |  |  |
| 0-0.09 | d.s. | d.s. | d.s. | d.s. | d.s. |
| >0.09-0.64 | 11 | 2.64 (0.91–7.70) | d.s. | d.s. | 1.65 (0.19–14.19) |
| >0.64-2.30 | 11 | 2.60 (0.88–7.71) | d.s. | d.s. | 1.76 (0.20–15.39) |
| >2.30-12.08 | 21 | 3.92 (1.38–11.19) | d.s. | d.s. | 2.99 (0.34–26.59) |
| >12.08 | d.s. | d.s. | d.s. | d.s. | d.s. |
| p-trend |  | 0.01 |  | 0.57 | 0.19 |
| **AIE AN Exposure^d.^** |  |  |  |  |  |
| 0-0.37 | 12 | 2.14 (0.79–5.82) | d.s. | d.s. | 1.01 (0.14–7.23) |
| >0.135-1.46 | 39 | 2.58 (1.00–6.66) | d.s. | d.s. | 1.85 (0.26–12.89) |
| p-trend |  | 0.06 |  | 0.89 | 0.41 |

d.s. Data suppressed to comply with NCI-UPitt data transfer agreement

1. RRs adjusted for race, sex, age, calendar time, salary/wage classification
2. Baseline category for RRs
3. Cumulative AN exposure, ppm-years (lagged 10 years)
4. Average intensity of AN exposure ppm (lagged 10 years)
