# Supplementary material for: A reevaluation of selected mortality risks in the updated NCI/NIOSH acrylonitrile cohort study
Source: Front Public Health. 2023 Apr 6;11:1122346. doi: 10.3389/fpubh.2023.1122346 (PMC10117843; doi:10.3389/fpubh.2023.1122346)
Supplement: Supplementary file 1 [file Data_Sheet_1.zip › Supplementary Material/Table 7.DOCX]

**Supplemental Table 7**

**UPitt Lung and Bronchus Cancer Relative Risks (RR) in Relation to AN Exposure Adjusted for Potential Confounding by Smoking Using Richardson’s Method, Plant 8, 1942-2011**

|  | **Unadjusted Lung and**  **Bronchus Cancer** | | **Chronic Obstructive Pulmonary Disease (COPD)** | | **Adjusted Lung and Bronchus Cancer** |
| --- | --- | --- | --- | --- | --- |
|  | **Obs** | **RR^a.^ (95%) CI** | **Obs** | **RR^a.^ (95%) CI** | **RR ^a.^ (95%) CI** |
| **Unexposed^b.^** | 73 | 1.0 | 40 | 1.0 | 1.0 |
| **Exposed** | 116 | 1.19 (0.87–1.63) | 51 | 0.91 (0.59–1.42) | 1.31 (0.76–2.24) |
| **Cum AN Exposure^c.^** |  |  |  |  |  |
| 0-0.09 | 18 | 1.13 (0.66–1.93) | 10 | 1.11 (0.54–2.31) | 1.01 (0.41–2.50) |
| >0.09-0.64 | 27 | 1.17 (0.74–1.85) | 13 | 0.98 (0.51–1.89) | 1.19 (0.53–2.65) |
| >0.64-2.30 | 33 | 1.13 (0.73–1.74) | 13 | 0.79 (0.41–1.52) | 1.42 (0.65–3.10) |
| >2.30-12.08 | 32 | 1.27 (0.83–1.95) | 13 | 0.89 (0.47–1.68) | 1.43 (0.66–3.10) |
| >12.08 | d.s. | d.s. | d.s | d.s. | d.s. |
| p-trend |  | 0.21 |  | 0.50 | 0.21 |
| **AIE AN Exposure^d.^** |  |  |  |  |  |
| 0-0.37 | 53 | 1.23 (0.83–1.81) | 25 | 1.01 (0.58–1.75) | 1.22 (0.62–2.39) |
| >0.135-1.46 | 52 | 1.12 (0.78–1.61) | 22 | 0.83 (0.49–1.42) | 1.35 (0.71–2.58) |
| >1.46 | 11 | 1.54 (0.80–2.94) | d.s. | d.s. | 1.52 (0.44–5.19) |
| p-trend |  | 0.29 |  | 0.60 | 0.30 |

d.s. Data suppressed to comply with NCI-UPitt data transfer agreement

1. RRs adjusted for race, sex, age, calendar time, salary/wage classification
2. Baseline category for RRs
3. Cumulative AN exposure, ppm-years (lagged 10 years)
4. Average intensity of AN exposure ppm (lagged 10 years)
