# Supplementary material for: A reevaluation of selected mortality risks in the updated NCI/NIOSH acrylonitrile cohort study
Source: Front Public Health. 2023 Apr 6;11:1122346. doi: 10.3389/fpubh.2023.1122346 (PMC10117843; doi:10.3389/fpubh.2023.1122346)
Supplement: Supplementary file 1 [file Data_Sheet_1.zip › Supplementary Material/Table 8.DOCX]

**Supplemental Table 8A**

**Details of the Log-Linear-Quadratic Models Used to Adjust Lung Cancer Mortality for Confounding by Smoking, Full Cohort**

| Model | Term ^b.^ | RR (categorical) | Model Estimate | Std. Error | p-value | RR  (continuous) | RR Combined Terms  (continuous) |
| --- | --- | --- | --- | --- | --- | --- | --- |
| Lung Cancer Unadjusted ^a.^ | AN Linear | - | 0.0622 | 0.0882 | 0.481 | 1.064 | 1.065 |
|  | AN Quadratic | - | 0.0010 | 0.0228 | 0.966 | 1.001 |  |
|  | Sex |  |  |  |  |  |  |
|  | Female | 1.00 | - | - | - | - |  |
|  | Male | 1.71 | 0.5366 | 0.1093 | <0.001 | - |  |
|  | Race |  |  |  |  |  |  |
|  | Nonwhite | 1.00 | - | - | - | - |  |
|  | White | 0.939 | -0.0632 | 0.1321 | <0.001 | - |  |
|  | Pay Type |  |  |  |  |  |  |
|  | Salary | 1.0 | - | - | - | - |  |
|  | Wage | 1.55 | 0.439 | 0.085 | <0.001 | - |  |
| COPD^c.^ | AN Linear | - | 0.0399 | 0.14 | 0.776 | 1.041 | 1.035 |
|  | AN Quadratic | - | -0.0058 | 0.0365 | 0.874 | 0.994 |  |
|  | Sex |  |  |  |  |  |  |
|  | Female | 1.00 | - | - | - | - |  |
|  | Male | 1.59 | 0.4586 | 0.1598 | 0.004 | - |  |
|  | Race |  |  |  |  |  |  |
|  | Nonwhite | 1.00 | - | - | - | - |  |
|  | White | 1.49 | 0.3927 | 0.2596 | 0.130 | - |  |
|  | Pay Type |  |  |  |  |  |  |
|  | Salary | 1.0 | - | - | - | - |  |
|  | Wage | 2.32 | 0.8400 | 0.1449 | <0.001 | - |  |
| Lung Cancer Adjusted | AN Linear | - | 0.0223 | 0.1655 | 0.893 | 0.0223 | 1.029 |
|  | AN Quadratic | - | 0.007 | 0.0431 | 0.875 | 0.0068 |  |
|  | Sex |  |  |  |  |  |  |
|  | Female | 1.00 | - | - | - | - |  |
|  | Male | 1.08 | 0.0780 | 0.1936 | 0.687 | - |  |
|  | Race |  |  |  |  |  |  |
|  | Nonwhite | 1.00 | - | - | - | - |  |
|  | White | 0.64 | -0.4559 | 0.2913 | 0.118 | - |  |
|  | Pay Type |  |  |  |  |  |  |
|  | Salary | 1.0 | - | - | - | - |  |
|  | Wage | 0.67 | -0.4007 | 0.1682 | 0.014 | - |  |

1. Similar to Figure 1 in Koutros et al. (2019)
2. AN linear and quadratic terms are ln(1 + cumulative AN exposure lagged 10 years (ppm-years))
3. Negative control health outcome used in Richardson method
