# Supplementary material for: A reevaluation of selected mortality risks in the updated NCI/NIOSH acrylonitrile cohort study
Source: Front Public Health. 2023 Apr 6;11:1122346. doi: 10.3389/fpubh.2023.1122346 (PMC10117843; doi:10.3389/fpubh.2023.1122346)
Supplement: Supplementary file 1 [file Data_Sheet_1.zip › Supplementary Material/Table 9.DOCX]

**Supplemental Table 8B**

**Details of the Log-Linear-Quadratic Models Used to Adjust Lung Cancer Mortality for Confounding by Smoking, Full Cohort (omitting Plant 4)**

| Model | Term ^b.^ | RR (categorical) | Model Estimate | Std. Error | p-value | RR  (continuous) | RR Combined Terms  (continuous) |
| --- | --- | --- | --- | --- | --- | --- | --- |
| Lung Cancer Unadjusted ^a.^ | AN Linear | - | 0.0985 | 0.0968 | 0.309 | 1.104 | 1.083 |
|  | AN Quadratic | - | -0.0186 | 0.0256 | 0.468 | 0.982 |  |
|  | Sex |  |  |  |  |  |  |
|  | Female | 1.00 | - | - | - | - |  |
|  | Male | 1.78 | 0.5753 | 0.1231 | <0.001 | - |  |
|  | Race |  |  |  |  |  |  |
|  | Nonwhite | 1.00 | - | - | - | - |  |
|  | White | 0.99 | -0.0099 | 0.1539 | 0.947 | - |  |
|  | Pay Type |  |  |  |  |  |  |
|  | Salary | 1.0 | - | - | - | - |  |
|  | Wage | 1.64 | 0.4924 | 0.0914 | <0.001 | - |  |
| COPD^c.^ | AN Linear | - | 0.0398 | 0.155 | 0.797 | 1.041 | 1.026 |
|  | AN Quadratic | - | -0.0138 | 0.0412 | 0.737 | 0.986 |  |
|  | Sex |  |  |  |  |  |  |
|  | Female | 1.00 | - | - | - | - |  |
|  | Male | 1.41 | 0.3459 | 0.1724 | 0.045 | - |  |
|  | Race |  |  |  |  |  |  |
|  | Nonwhite | 1.00 | - | - | - | - |  |
|  | White | 1.40 | 0.332 | 0.288 | 0.249 | - |  |
|  | Pay Type |  |  |  |  |  |  |
|  | Salary | 1.0 | - | - | - | - |  |
|  | Wage | 2.16 | 0.7698 | 0.1526 | <0.001 | - |  |
| Lung Cancer Adjusted | AN Linear | - | 0.0587 | 0.1826 | 0.748 | 1.060 | 1.0001 |
|  | AN Quadratic | - | -0.0048 | 0.0485 | 0.921 | 0.995 |  |
|  | Sex |  |  |  |  |  |  |
|  | Female | 1.00 | - | - | - | - |  |
|  | Male | 1.26 | 0.2294 | 0.2119 | 0.279 | - |  |
|  | Race |  |  |  |  |  |  |
|  | Nonwhite | 1.00 | - | - | - | - |  |
|  | White | 0.71 | -0.342 | 0.3265 | 0.295 | - |  |
|  | Pay Type |  |  |  |  |  |  |
|  | Salary | 1.0 | - | - | - | - |  |
|  | Wage | 0.76 | -0.2775 | 0.1779 | 0.1188 | - |  |

1. Similar to Figure 1 in Koutros et al. (2019)
2. AN linear and quadratic terms are ln(1 + cumulative AN exposure lagged 10 years (ppm-years))
3. Negative control health outcome used in Richardson method
